# Suppressing ERK Pathway Impairs Glycochenodeoxycholate-Mediated Survival and Drug-Resistance in Hepatocellular Carcinoma Cells

**DOI:** 10.3389/fonc.2021.663944

**Published:** 2021-07-13

**Authors:** Bingxin Li, Maojun Zhou, Jue Wang, Hongjuan Xu, Manyi Yang

**Affiliations:** ^1^ Department of Hepatobiliary and Pancreatic Surgery, National Health Commission (NHC) Key Laboratory of Nanobiological Technology, Xiangya Hospital, Central South University, Changsha, China; ^2^ Department of Oncology, NHC Key Laboratory of Cancer Proteomics, National Center for Geriatrics Clinical Research, State Local Joint Engineering Laboratory for Anticancer Drugs, Xiangya Hospital, Central South University, Changsha, China

**Keywords:** hepatocellular carcinoma cells, glycochenodeoxycholate, extracellular signal-regulated kinase 1/2, anti-apoptosis proteins, pro-apoptotic proteins

## Abstract

Glycochenodeoxycholate (GCDA), a toxic component in bile salts, is involved in carcinogenesis of gastrointestinal tumors. The objective of this research was to study the function of ERK1/2 in the GCDA-mediated survival and drug-resistance in hepatocellular carcinoma cells (HCCs). Firstly, extracellular signal-regulated kinase 1/2 (ERK1/2) was detected extensively expressed in liver cancer cells, and silencing ERK1/2 by RNA interference could suppress GCDA-stimulated survival and promote apoptosis. Furthermore, phosphorylation of endogenous ERK1/2 could be potently stimulated by GCDA in combination with enhanced chemoresistance in QGY-7703 hepatocellular carcinoma cells. The GCDA-mediated proliferation and chemoresistance could be impaired by PD98059, which acted as an inhibitor to block the phosphorylation of ERK1/2. Mechanistically, PD98059 was able to potently suppress GCDA-stimulated nuclear aggregation of ERK1/2 and p-ERK1/2, upregulate pro-survival protein Mcl-1 and downregulate pro-apoptotic protein Bim. The results of this study indicated that disruption of ERK1/2 by blocking phosphorylation or nuclear translocation may put forward new methods for solving the problem of GCDA-related proliferation and drug-resistance in liver cancer treatment.

## Introduction

Hepatocellular carcinoma (HCC) is the most common liver cancer nowadays, and more than 700,000 cases are diagnosed every year (1). The pathogenesis of HCC is extremely complex, but evolving information suggests that the major risk factors for HCC in contemporary clinical practice include alcoholism, non-alcoholic fatty liver disease (NFLD), hepatitis B virus (HBV), and hepatitis C virus (HCV) (2, 3). Most patients with advanced liver cancer will choose chemotherapy. However, patients with HCC usually develop resistance to 5-fluorouracil, doxorubicin or cisplatin, which are the traditional chemotherapeutics. Unfortunately, sorafenib, the new generation of drugs, did not achieve the desired results (4). Thus, it is very important to explore the resistance mechanism of HCC.

Bile salts are the major ingredients in bile, which are secreted by liver cells and involved in fat digestion and absorption. Glycochenodeoxycholate (glycine conjugate of chenodeoxycholate, GCDA), a toxic component in bile salts, is involved in carcinogenesis of gastrointestinal tumors (5). Previous researches have indicated that GCDA could stimulate the growth of Barrett’s adenocarcinoma cells and non-neoplastic Barrett cell lines through PI3 kinase/Akt pathway and p38/ERK/MAPK pathway respectively (6, 7). Satoshi et al. (8) found that glycochenodeoxycholate acid could promote the proliferation of intestinal epithelia *via* decreasing cyclic AMP and increasing histone H2AX phosphorylation after exposure to *γ*-rays. Another study demonstrated that the biliary tract cancer could be induced by GCDA *via* aggregation of 8-OHdG and oxidative DNA damage (9). The metabolic disorder of bile salts could lead to abnormal bile salt accumulation; it could be a direct factor in the development of HCC. A study by Wang et al. (10) found that GCDA might upregulate pro-survival proteins (Mcl-1, Survivin, and Bcl-2) and eventually results in chemoresistance of HCC cells. However, the specific intracellular mechanism of GCDA-mediated hepatocellular carcinoma development remains to be further studied.

As a member of the mitogen activated protein kinase family, the extracellular signal-regulated kinase (ERK) takes a key part in transmitting signals from receptors on the cell surface into the nucleus (11). Signals transmitted from MEK1/2 can phosphorylate ERK1/2 at Thr and Tyr residues (12). Then the activated ERK1/2 phosphorylates downstream substrates and eventually causes cell proliferation, differentiation, and canceration (13). Usually, ERK1/2 is mainly distributed in the cytoplasm of normal cells. Upon stimulation, many ERK1/2 molecules shift to the nucleus, Golgi, mitochondria, endosomes/lysosomes and endoplasmic reticulum (14). The main translocation seems to be the entry into the nucleus, which is an important place for signal transmission downstream of ERK (13). Because the nuclear translocation of ERK is mainly important for cell proliferation, prevention of such translocation can be used as a novel strategy to combat cancer (15). Furthermore, ERK1/2 signaling is an important regulator of cell-intrinsic Bcl-2-regulated apoptotic signaling (16). In most situations, ERK1/2 signaling accelerates cell growth *via* stimulating anti-apoptosis proteins (Bcl-2, Mcl-1, and Bcl-xL) and inhibiting pro-apoptotic proteins (Bim, Bad, Bmf, and Puma) (14). Thus, suppression of ERK1/2 pathway in tumor cells might serve as an effective way to prevent cancer development.

The chemoresistance of ERK1/2 has been extensively studied in other cancers. In radioresistant glioblastoma multiforme cells, cell survival could be promoted through ERK1/2 signaling when pSTAT3(Y705) was inhibited (17). ERK1/2 and p38 MAPK signaling pathways were significantly involved in neoplastic transformation and cisplatin-resistance in nasopharyngeal carcinoma cell lines (18). However, there was little in-depth research for the chemoresistance of ERK1/2 in HCC. A published study has shown that the activation of ERK1/2 could decrease the sensitivity to sorafenib in the HCC cells (Bel-7402 and SMMC-7721) (19). Our previous studies have confirmed the association of GCDA with drug resistance in HCC cells (10, 20). But the exact function of ERK1/2 in such process has not been clarified. In this research, we proved that GCDA mediates activation and nuclear accumulation of ERK1/2, which finally results in promoting anti-apoptotic function in human liver cancer cells.

## Materials and Methods

### Cell Culture

LO2, HepG2, Bel-7402, Bel-7404, SMMC-7721, Huh7, MHC97-H, and QGY-7703 HCC cell lines were originally from the Institute of Biochemistry and Cell Biology (CAS, Shanghai, China). LO2 and Bel-7402 cell line were maintained in RPMI-1640 medium (Thermo Fisher Scientific, Waltham, USA) with 10% fetal bovine serum (ExCell Bio, Shanghai, China). HepG2, Bel-7404, SMMC-7721, Huh7, MHC97-H, and SMMC-7721 QGY-7703 cell lines were cultivated in Dulbecco’s modified Eagle’s medium (Hyclone, Logan, USA) supplemented 10% FBS. Cell lines were incubated at 37°C with 5% CO_2_.

### Reagents and Antibodies

The antibodies of ERK1 + ERK2 and ERK1 (pT202/pY204) + ERK2 (pT185/pT187) were obtained from Abcam (Cambridge, UK). Goat-anti rabbit HRP antibody and anti-GAPDH antibody were from Cell Signaling Technology (Danvers, MA, USA). PD98059, a specific inhibitor of ERK kinase, was from Calbiochem (San Diego, CA, USA). Glycochenodeoxycholate (GCDA) and cisplatin were obtained from Sigma-Aldrich (St. Louis, USA). 5-Fluorouracil (5-FU) was purchased from Xudong Haipu Pharmaceutical (Shanghai, China). The Annexin V-FITC apoptosis detection kit was purchased from Becton, Dickinson and Company (BD, Franklin Lake, NJ).

### siRNA and Transfections

For RNA interference, siRNA 225 (ACACGCAGUUGCAGUACAU), 888 (GACCGGAUGUUAACCUUUA), and 933 (GAAACUACCUACAGUCUCU) targeting human ERK1, siRNA 355 (GUGCUCUGCUUAUGAUAAU), 513 (CACCAACCAUCGAGCAAAU) and 714 (CCACCUGUGAUCUCAAGAU) targeting human ERK2 and negative control siRNA (UUCUCCGAACGUGUCACGU) were from Shanghai Gene Pharma, Co., Ltd (Shanghai, China). QGY-7703 cells were transfected with siRNAs for 24 h using Lipofectamine RNAi max (Invitrogen, NY, USA).

### CCK8 Assay

QGY-7703 cells were seeded in 96 well plates. Then GCDA, drugs, or inhibitors were used to treat cells. After various treatments, each well was supplemented with 10 μl of CCK8 solution and incubated for 1.5 h. After that, the absorbance was determined by microplate microscopy at 450 nm (BioTek, Winooski, VT).

### Western Blot Analysis

The samples of QGY-7703 cells were lysed with detergent buffer for 30 min on ice. Then cell products were scraped from the wells and centrifuged for 15 min at 12,000 rpm. Protein, 30 μg, was loaded onto 10% SDS-PAGE and transferred to a polyvinylidene difluoride (PVDF) membrane. After blocking with blocking solution for 2 h at room temperature, cells were then incubated at 4°C overnight with primary antibodies, followed by washing with 1× TBST and incubating with horseradish peroxidase-conjugated anti-mouse or anti-rabbit secondary antibodies (1:5,000) with shaking for 1 h. Results were detected using WesternBright™ ECL (Advansta, USA), and the bands were scanned and quantified using the FluorChem FC3 system.

### Flow Cytometry

QGY-7703 cells were transfected with siRNA888 and siRNA513 together for 24 h. Following treatment with 100 µM GCDA, cells were collected and washed with cold PBS. After resuspending with 1× binding buffer, 3 μl Annexin V-FITC and propidium iodide (PI) (Becton, Dickinson and Company, NJ) were used to treat the cells for 15 min. The apoptotic rate was detected by flow cytometry.

### Immunofluorescence

In 24-well plates, QGY-7703 cells were cultured with a glass coverslip overnight. After cells were exposed to GCDA or GCDA + PD98059 for 8 h, 4% paraformaldehyde was used to fix cells for 15 min. The cells were washed with TBST and performed using ERK1/2 or p-ERK1/2 antibody at 4°C overnight after incubating with Alexa Fluor^®^594 goat antibody at 37°C for 1 h. Cell nuclei were stained with DAPI for 2 min. At last, the results were photographed with a fluorescence microscope.

### Statistical Analysis

SPSS software V17.0 was used to perform the statistical analysis. All data were displayed as the means ± SD. Inter-group differences were assessed by Student’s t-test. P <0.05 was the considered level of statistical significance.

## Results

### ERK1/2 Acts a Part in GCDA-Induced Survival of Human Liver Carcinoma Cells

**Figure 1:**
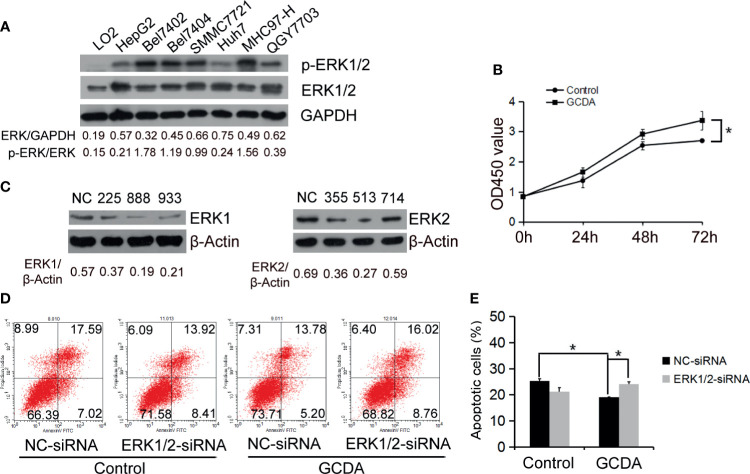
ERK1/2 act a part in GCDA-mediated survival of human liver carcinoma cells. **(A)** Expression of ERK1/2 in normal liver cells (LO2) and seven liver carcinomas cell lines (HepG2, Bel7402, Bel7404, SMMC7721, Huh7, MHC97-H and QGY7703) was detected by western blot and quantified by Alphaview software. **(B)** GCDA (100µM) was used to treat QGY7703 cells for 0h, 24h, 48h and 72h. CCK8 was performed to determine the viable cells. **(C)** QGY7703 cells were transfected with siRNA targeting ERK1 (225, 888 and 933) and ERK2 (355, 513 and 714). After 24h, whole cell extracts were analyzed by western blot using ERK1 and ERK2 antibodies. NC, negative; control siRNA. **(D, E)** siRNA888 targeting ERK1 and siRNA513 targeting ERK2 were transfected into QGY7703 cells together. 24 hours later, 100μM GCDA was used to treat cells for 24h. Apoptosis were determined using flow cytometry. All data represent the mean±SD and were obtained from at least three independent experiments. *P < 0.05, (Student’s t-test).

The ERK1/2 cascade is best known for its role in proliferation, differentiation, and tumorigenesis (13). Firstly, we measured the endogenous protein levels of ERK1/2 in normal liver cells (LO2) and seven HCC cell lines (HepG2, Bel-7402, Bel-7404, SMMC7721, Huh7, MHC97-H, and QGY-7703. The result of Western blot showed that ERK1/2 was extensively expressed in all the liver cancer cells we detected (**Figure 1A**). Next, to test whether GCDA promoted HCC cell proliferation, we treated QGY-7703 cell line with 100 μM GCDA for 0, 24, 48, and 72 h, and then checked the viable cells by CCK8. Results indicated that viable cells significantly increased after treatment with GCDA for72 h (**Figure 1B**).

To determine whether ERK1/2 affected the GCDA-induced survival of HCC cells, we designed siRNAs targeting ERK1 (225, 888, and 933) and ERK2 (355, 513, and 714). All the siRNAs were transfected into QGY-7703 cells. Then immunoblotting was done to determine the interference efficiency. As shown in **Figure 1C**, ERK1 and ERK2 protein expressions were inhibited by siRNA888 and siRNA513, respectively. After siRNA888 targeting, ERK1 and siRNA513 targeting, ERK2 was transfected into QGY-7703 cell line together; GCDA was used to treat the cells for 24 h. Apoptotic cells were analyzed using annexin V binding on FASC. Flow cytometry results demonstrated that GCDA could repress apoptosis. But after ERK1/2 was silenced, the apoptotic cells were increased (**Figures 1D, E**). In other words, specific depletion of ERK1/2 blocked GCDA-stimulated cell survival. These results indicated that ERK1 and ERK2 molecules have played a role in the survival of hepatoma cells mediated by GCDA.

### GCDA Induces ERK1/2 Phosphorylation, Which May Be Involved in Prolonged Survival of Human Liver Cancer Cells

**Figure 2:**
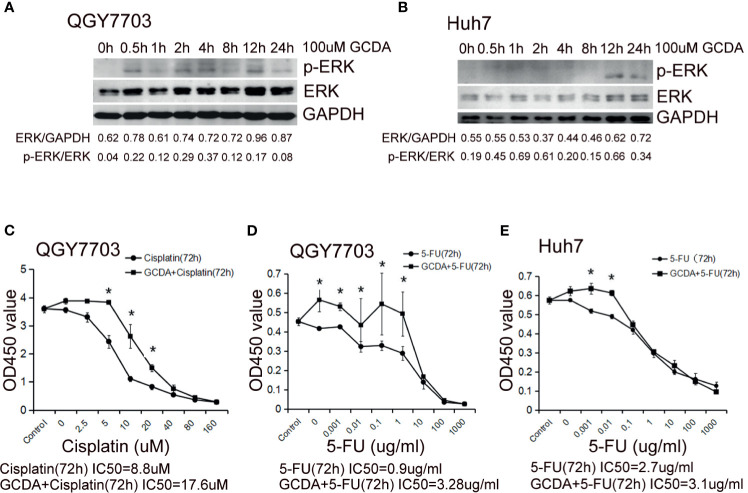
GCDA induces ERK1/2 phosphorylation, which may be involved in prolonged survival of human liver cancer cells. **(A, B)** 100μM GCDA was used to treat QGY7703 and Huh7 cells for 0h, 0.5h, 1h, 2h, 4h, 8h, 12h and 24h. The expression level of ERK1/2 and p-ERK1/2 were tested by western blot and quantified by Alphaview software. **(C–E)** Antitumor drug (Cisplatin or 5-FU) or GCDA (100μM)+antitumor drug(Cisplatin or 5-FU) were used to treat QGY-7703 and Huh7 cells for 72h. CCK8 was performed to determine the viable cells. IC50 is calculated as the concentration of Cisplatin or 5-FU inducing a 50% reduction in cell viability. All data represent the mean±SD and were obtained from at least three independent experiments. *P < 0.05 (Student’s t-test).

Furthermore, we investigated potential mechanisms involved in the GCDA-induced HCC cell survival. QGY-7703 and Huh7 cells were treated with 100 μM GCDA for 0, 0.5, 1, 2, 4, 8, 12, and 24 h. Results demonstrated that the activated ERK1/2 increased obviously after GCDA treatment in QGY-7703 and Huh7 cells, while the expression of endogenous ERK1/2 changed little (**Figures 2A, B**).

Cisplatin has been known as one of the most potential and widely used drugs, which is effective in a variety of solid cancers such as testicular, ovarian, head and neck, bladder, lung, cervical, melanoma, and lymphomas (21–25). The antimetabolite 5-fluorouracil (5-FU), which can inhibit thymidylate synthase, is a widely used antitumor agent (26). In order to check the effect of GCDA-induced ERK1/2 activation on cell survival, QGY-7703 and Huh7 cells were treated with antitumor drug (cisplatin or 5-FU) or GCDA (100 μM) + antitumor drug (cisplatin or 5-FU) for 72 h. The IC50 of cisplatin for QGY-7703 is 8.8 µM (**Figure 2C**). The IC50 value of 5-FU is 0.9 µg/ml for QGY-7703 and 2.7 µg/ml for Huh7, respectively (**Figures 2D, E**). However, following GCDA treatment, the IC50 concentrations were increased obviously. Such results indicate that GCDA can significantly enhance resistance to drugs. Therefore, we speculated the involvement of activated ERK1/2 in chemoresistance induced by GCDA.

### The MAPK/ERK1/2 Inhibitor PD98059 Decreases GCDA-Stimulated Cell Proliferation

**Figure 3:**
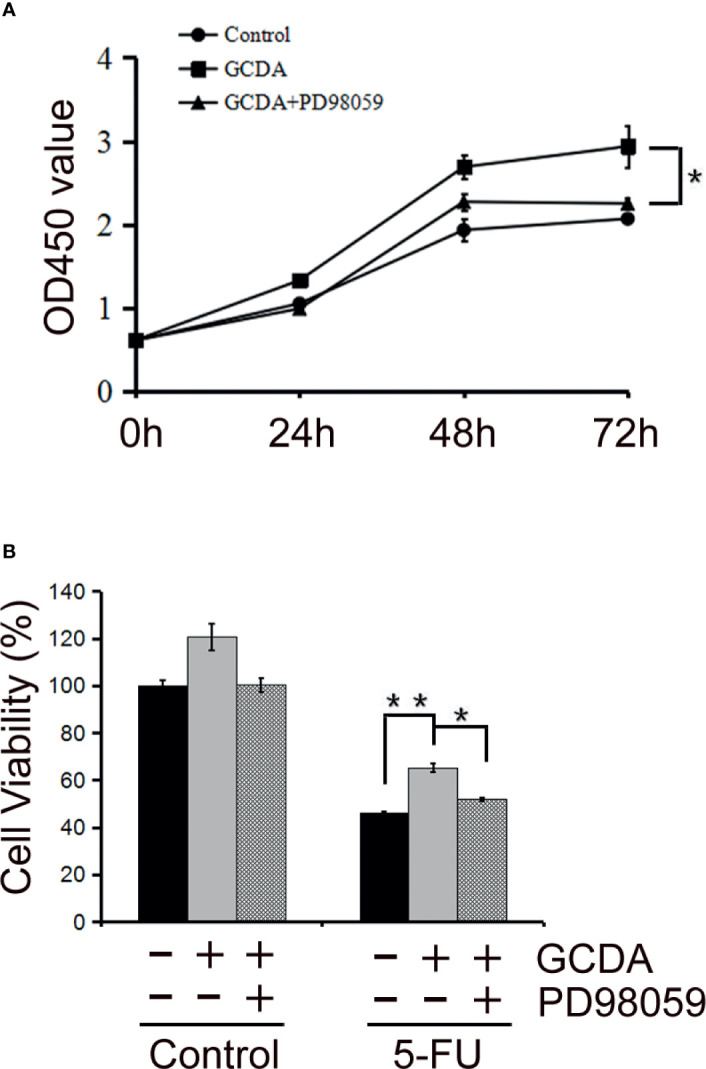
PD98059, the ERK1/2 inhibitor, attenuates GCDA-mediated survival and drug-resistance in HCC cells. **(A)** PD98059 could inhibit phosphorylation of ERK1/2. QGY-7703 cells were preincubated with PD98059 (10μM) for 0.5 h, followed by treatment with 100μM GCDA for 24h, 48h and 72h. CCK8 was performed to determine the viable cells.**(B)** QGY7703 cells were treated with or without PD98059 (10μM) for 0.5 h, followed by treatment with GCDA (100μM) or GCDA(100μM) +antitumor drug (1μg/ml 5-FU) for 72h. Then CCK8 was performed to determine the viable cells. Data in graphs are as mean±SD. All experiments data were repeated at least three independent experiments. *P < 0.05, **P < 0.01 (Student’s t-test).

To further verify the role of activated ERK1/2 in HCC cells, the MAPK/ERK1/2 inhibitor PD98059, which could inhibit phosphorylation of ERK1/2, was used (27). We treated QGY-7703 cells with GCDA (100 μM) or GCDA (100 μM) + PD98059 (10 μM) for 24, 48, and 72 h. Then, CCK8 was done to test the viability of QGY-7703 cells. CCK8 experiments showed that suppression of ERK1/2 activation by PD98059 would decrease proliferation of liver cancer cells (**Figure 3A**). Next, QGY-7703 cells were treated with or without PD98059 (10 μM) for 0.5 h, followed by treatment with GCDA (100 μM) or GCDA (100 μM) + antitumor drug (1 μg/ml 5-FU) for 72 h. Results of CCK8 showed that PD98059 significantly attenuated the chemoresistance induced by GCDA, which could prolong cell survival following treatment with 5-FU (**Figure 3B**). In conclusion, these findings implied that phosphorylation (or activation) of ERK1/2, which is attenuated by PD98059, is important for the survival and chemoresistance of GCDA-mediated HCC cells.

### PD98059 Suppresses GCDA-Induced Nuclear Aggregation of ERK1/2 and p-ERK1/2

**Figure 4:**
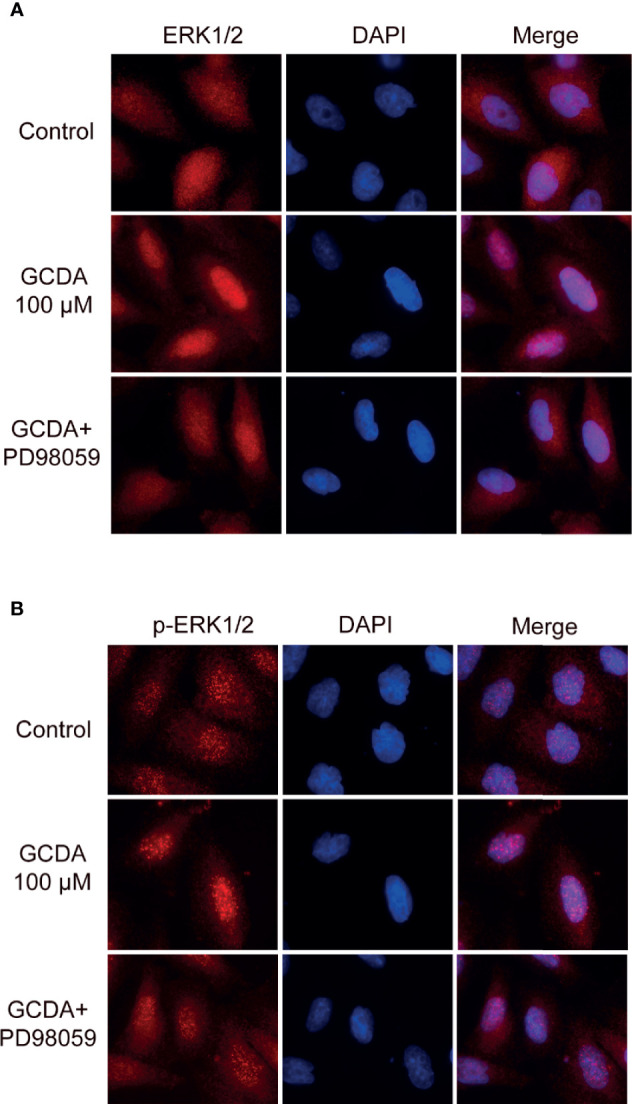
PD98059 suppresses GCDA-induced nuclear aggregation of ERK1/2 and p-ERK1/2. **(A, B)** QGY-7703 cells were preincubated with PD98059 (10μM) for 0.5 h, followed by treatment with 100μM GCDA for 8h. Immunofluorescence staining was done using ERK1/2 or p-ERK1/2 antibody. Cell nuclei were stained with DAPI for 2 min. The experiments were repeated three times.

In unstimulated cells, ERK1/2 molecules are usually located in the cytoplasm (15). Under stimulation, numerous ERK1/2 molecules are translocated to the nucleus (15). ERK1/2 localization plays a significant role in determining the strength of this pathway. Therefore, we examined the localization of ERK1/2 and p-ERK1/2 following GCDA (100 μM) or GCDA (100 μM) + PD98059 (10 μM) treatment. The results of immunofluorescence staining showed that ERK1/2 proteins were distributed in both cytoplasm and nucleus and more p-ERK1/2 proteins accumulated in the nucleus as small spots in resting HCC cells (**Figures 4A, B**). Following GCDA treatment, most ERK1/2 proteins gathered in the nucleus, while more p-ERK1/2 proteins accumulated in the nucleus as bigger speckles. However, after PD98059 treatment, the aggregation of ERK1/2 and p-ERK1/2 proteins in the nucleus significantly decreased (**Figures 4A, B**). Collectively, the above data suggested that nuclear accumulation of ERK1/2 and p-ERK1/2 induced by GCDA could be impaired by PD98059.

### PD98059 Restrains GCDA-Induced Increase of Mcl-1 and Decrease of Bim

**Figure 5:**
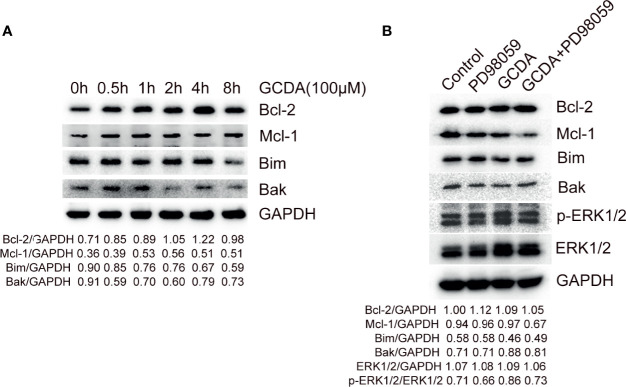
PD98059 suppresses GCDA-stimulated increase of Mcl-1 and decrease of Bim. **(A)** 100μM GCDA was used to treat QGY-7703 cells for 0h, 0.5h, 1h, 2h, 4h and 8h. Expression of Bcl-2, Mcl-1, Bim and Bak was tested by western blot and quantified by Alphaview software. **(B)** QGY-7703 cells were preincubated with 10μM PD98059 for 0.5 h, followed by treatment with 100μM GCDA for 8h. All cell extracts were analyzed using Bcl-2, Mcl-1, Bim, Bak, p-ERK1/2 and ERK1/2 antibodies by Western blotting and quantified by Alphaview.

ERK1/2 signaling has been verified to have the ability to regulate some members of the Bcl-2 family, which can contribute to tumor cell survival *via* increasing anti-apoptotic factors and decreasing pro-apoptotic members of Bcl-2 family (16). Hence, we inspected the level of some Bcl-2 family members following GCDA (which can activate ERK1/2 pathway) or PD98059 (which can repress ERK1/2 pathway) treatment. Firstly, 100 μM GCDA was used to treat QGY-7703 cells for 0, 0.5, 1, 2, 4, and 8 h. Immunoblot had been done to check the levels of Bcl-2, Mcl-1, Bim, and Bak. We observed that GCDA could promote expression of Bcl-2 and Mcl-1, both of which are anti-apoptotic Bcl-2 family members and decrease expression of Bim and Bak, both of which are pro-apoptotic Bcl-2 family members (**Figure 5A**). Next, in order to determine whether the suppression of ERK1/2 signaling regulated expression of Bcl-2 family members, GCDA (100 μM) or GCDA (100 μM) + PD98059 (10 μM) was used to treat QGY-7703 cells for 8 h. Results showed that inhibition of ERK1/2 by PD98059 could block GCDA-induced increase of Mcl-1 and decrease of Bim. However, Bcl-2 and Bak did not change significantly (**Figure 5B**). Our data supported the notion that GCDA might facilitate cell survival *via* regulation proteins of Bcl-2 family, some of which could be inhibited by PD98059. Such results indicated that activation of ERK1/2 pathway induced by GCDA could mediate certain members of the Bcl-2 family.

## Discussion

Glycochenodeoxycholate is one of the toxic bile salts and may promote HCC invasion *via* activation of autophagy (28, 29). In the current study, survival and chemoresistance to cisplatin and 5-FU induced by GCDA have been verified in QGY-7703 cell line (**Figures 1B** and **2C–E**).

The ERK1/2 signaling pathway is considered to have great effects on proliferation, invasion, and migration in cancer cells. Numerous studies have confirmed that ERK1/2 signaling is the main regulator that promotes the progression of human hepatocellular carcinoma (30–34). ERK1/2 participates in liver injury in human liver stem cells (35, 36). Also, the aggressive behavior of HCC cells has a positive relationship with the level of phosphorylated ERK and activated level of hepatic stellate cells (aHSCs) (37). Thus, we speculated GCDA mediated survival and chemoresistance *via* the ERK1/2 pathway in liver cancer cells. Our results showed that activation levels of ERK1/2 increased significantly following GCDA treatment in hepatocellular carcinoma cells (**Figures 2A, B**). After ERK1/2 was silenced by siRNA or phosphorylation of ERK1/2 was blocked by PD98059, cell proliferation was significantly decreased (**Figures 1D, E** and **3A**). In the light of those results, it is reasonable to suggest that the ERK1/2 pathway is involved with GCDA-induced survival in HCC cells.

Because of binding to many scaffold proteins or cytoplasmic anchors in resting cells, ERK1/2 is usually localized in the cytoplasm (15). Upon stimulation, numerous ERK1/2 molecules are translocated to the nucleus (14). In QGY-7703 cells, ERK1/2 and p-ERK1/2 could aggregate in the nucleus after treatment with GCDA (**Figures 4A, B**). Therefore, nuclear aggregation of ERK1/2 molecules must be relevant to HCC cell proliferation signal transduction following GCDA treatment. However, such nuclear accumulation could be decreased by inhibitor PD98059 (**Figures 4A, B**), which meant that the GCDA-induced survival signal is impaired by PD98059. Based on the evidence in this study, preventing ERK1/2 from entering the nucleus may be considered as a novel strategy to arrest liver cancer growth.

Activated ERK1/2 is also translocated to mitochondria, Golgi, the endoplasmic reticulum, or endosomes/lysosomes, thereby influencing cell physiology (38). Among them, the mitochondrial anchored ERK1/2 molecules are involved with the mitochondrial apoptosis pathway *via* affecting Bcl-2 family members (16). Usually, ERK1/2 signaling facilitates cell survival *via* activating pro-survival proteins (Bcl-2, Mcl-1, and Bcl-xL) and inhibiting pro-apoptotic proteins (Bim, Bad, Bmf, and Puma) (16). Among them, the transcription of pro-survival protein Bcl-2 can be promoted by ERK1/2 signaling through cAMP-responsive element-binding protein (CREB) (39). Besides, Bcl-2 itself can also be phosphorylated at Ser87 by ERK1/2, which is proposed to inhibit its pro-survival function (14). The mRNA level of Mcl-1 is verified to be promoted in response to ERK1/2 pathway *via* CREB or transcription factor ELK1 (40). Also, the short half-life of Mcl-1 protein can be prolonged *via* direct phosphorylation by ERK1/2 (41). Bim, is a prominent target of ERK1/2 signaling (42). ERK1/2-induced activation of Bim leads to ubiquitylation and degradation (43). Bak is the apoptotic effector protein of Bcl-2 family. Bak can be directly activated by Bim and cause the release of cytochrome c (44). In the present research, we observed that inhibiting ERK1/2 phosphorylation by PD98059 blocked GCDA-induced increase of Mcl-1 and decrease of Bim. However, Bcl-2 and Bak did not change significantly (**Figures 5A, B**). These results showed that the GCDA-induced change of Mcl-1 and Bim might be regulated by ERK1/2 pathway, while the variation of Bcl-2 and Bak may be induced by GCDA in an ERK-independent manner.

In conclusion, the present results found that GCDA-stimulated cell proliferation and chemoresistance could be attenuated *via* targeting the ERK pathway. GCDA was able to potently promote phosphorylation and nuclear aggregation of ERK1/2 molecules, which eventually led to the increased level of anti-apoptotic Bcl-2 family member proteins (Bcl-2 and Mcl-1) and the decreased expression of pro-apoptotic Bcl-2 family members (Bim and Bak). The inhibitor PD98059 not only suppressed the phosphorylation of ERK1/2, but also blocked ERK1/2 nuclear accumulation of the nucleus and attenuated GCDA-stimulated increase of Mcl-1 and decrease of Bim. Therefore, disruption of the pro-survival function of GCDA by blocking phosphorylation and nuclear accumulation of ERK1/2 molecules represents tactics for treating GCDA-related liver cancer and chemoresistance.

## Data Availability Statement

The original contributions presented in the study are included in the article/**Supplementary Material**. Further inquiries can be directed to the corresponding author.

## Author Contributions

MY contributed to conceive and designed the experiments. BL performed the data analyses and wrote the manuscript. MZ contributed significantly to analysis and manuscript preparation. JW and HX helped perform the analysis with constructive discussions. All authors contributed to the article and approved the submitted version.

## Funding

This work was supported by the National Natural Science Foundation of China (grant numbers 81703412, 81402001) and the Natural Science Foundation of Hunan Province (grant numbers 2020JJ4889, 2018JJ3830, and 2016JJ3177).

## Conflict of Interest

The authors declare that the research was conducted in the absence of any commercial or financial relationships that could be construed as a potential conflict of interest.
