# Supplementary figures and images for: Suppressing ERK Pathway Impairs Glycochenodeoxycholate-Mediated Survival and Drug-Resistance in Hepatocellular Carcinoma Cells
Source: Front Oncol. 2021 Jul 13;11:663944. doi: 10.3389/fonc.2021.663944 (PMC8313996; doi:10.3389/fonc.2021.663944)

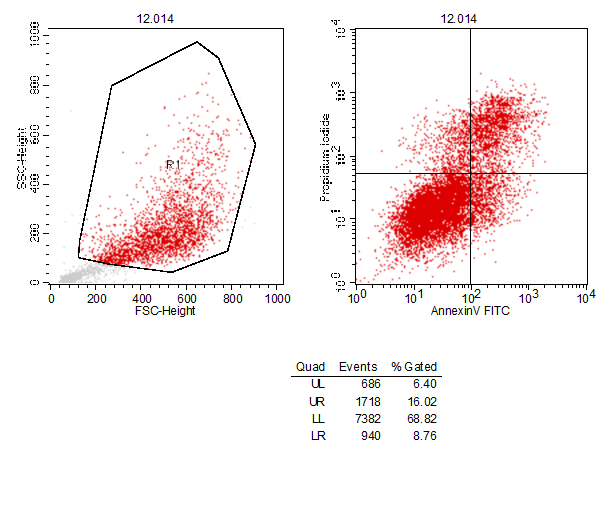

Supplement: Supplementary file 1 [file DataSheet_1.zip › Supplementary Material/Flow cytometry/GCDA-ERK siRNA.png]

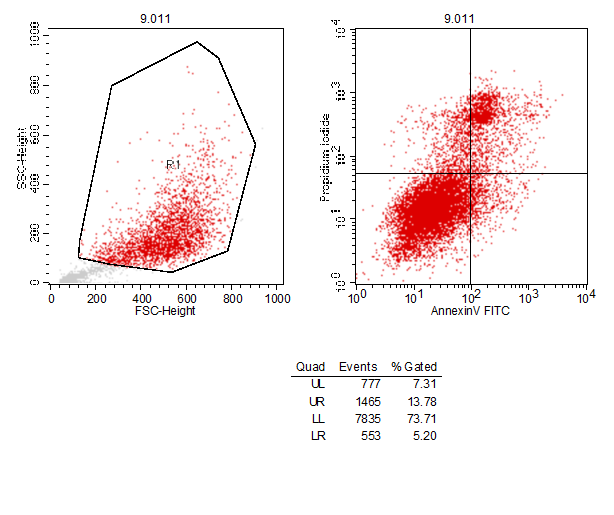

Supplement: Supplementary file 1 [file DataSheet_1.zip › Supplementary Material/Flow cytometry/GCDA-NC-siRNA.png]

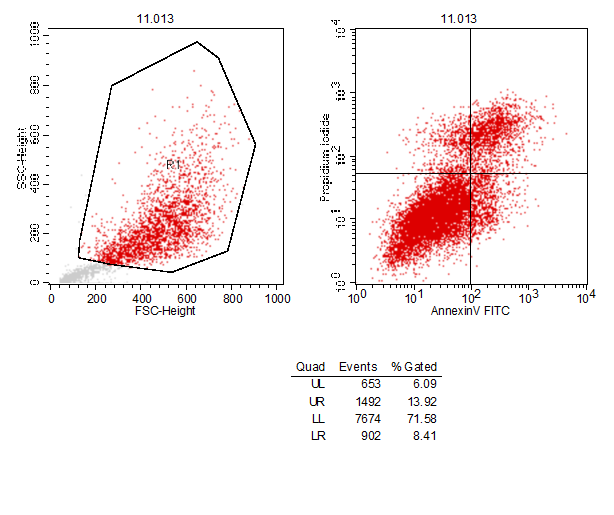

Supplement: Supplementary file 1 [file DataSheet_1.zip › Supplementary Material/Flow cytometry/control-ERK siRNA.png]

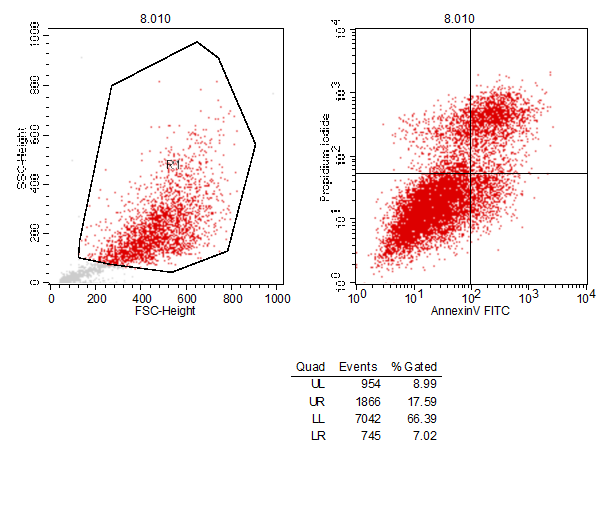

Supplement: Supplementary file 1 [file DataSheet_1.zip › Supplementary Material/Flow cytometry/control-NC-siRNA.png]

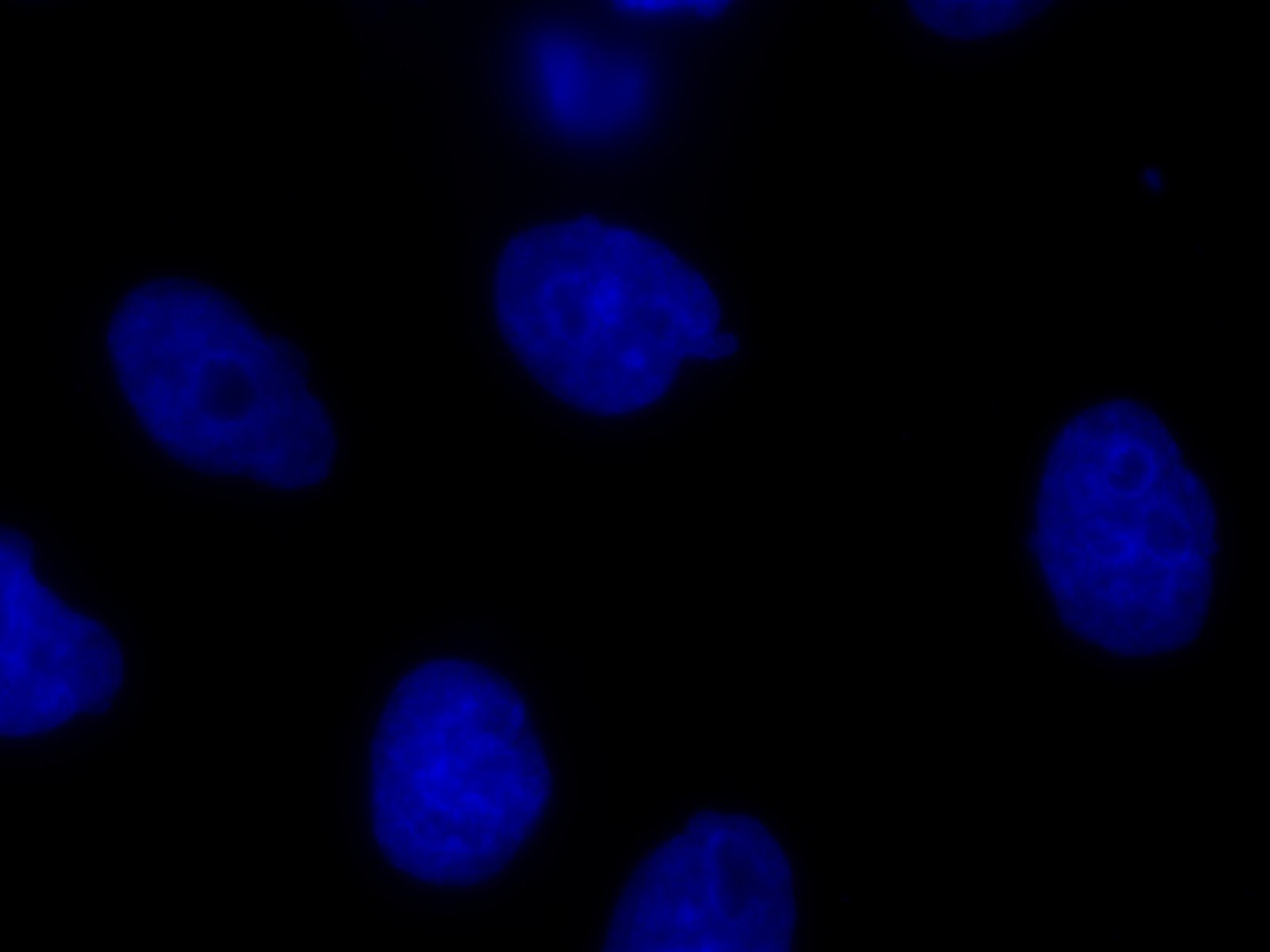

Supplement: Supplementary file 1 [file DataSheet_1.zip › Supplementary Material/IF/1.18-ERK+MITO Control 3 ^^/b.jpg]

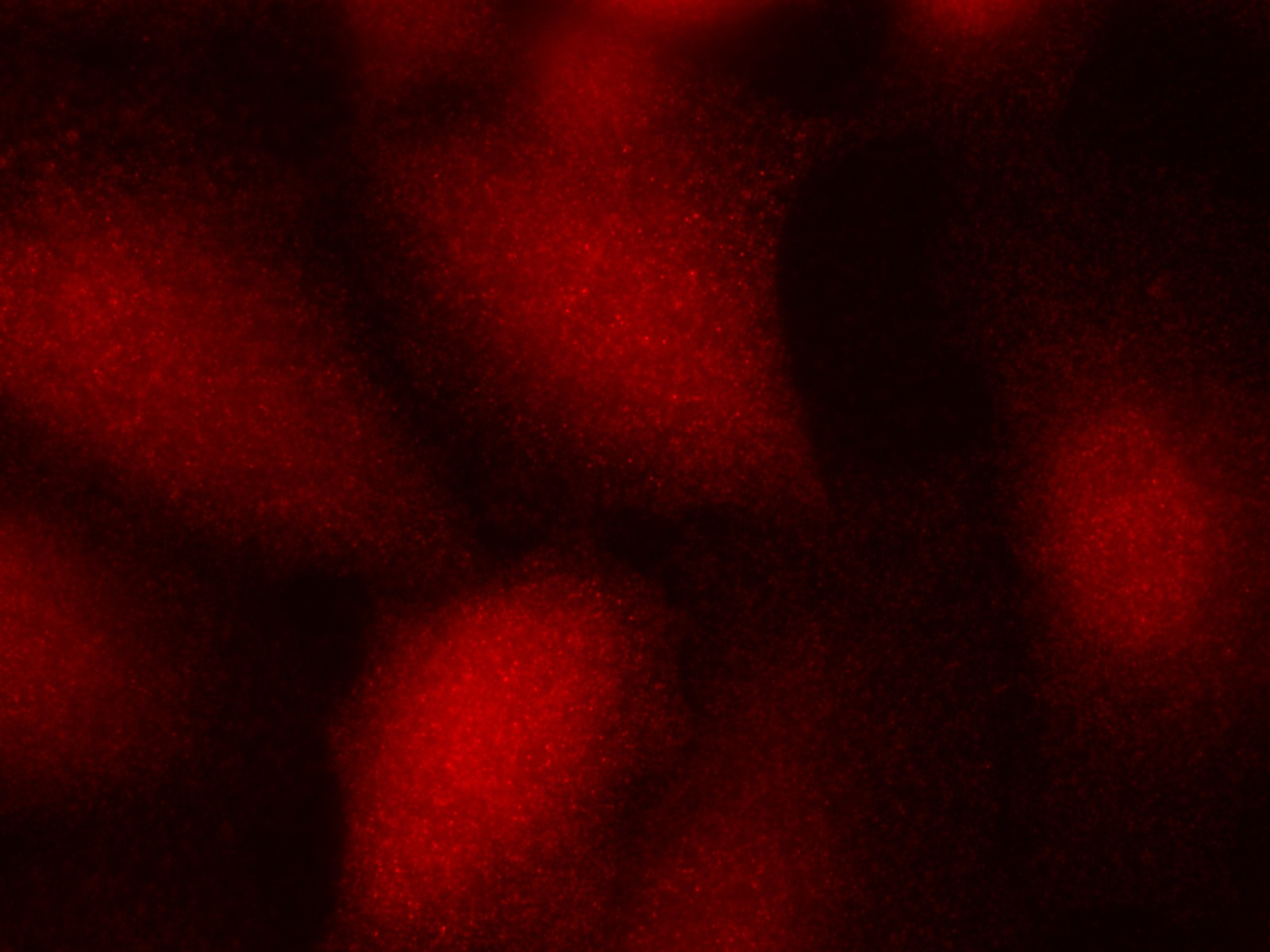

Supplement: Supplementary file 1 [file DataSheet_1.zip › Supplementary Material/IF/1.18-ERK+MITO Control 3 ^^/r.jpg]

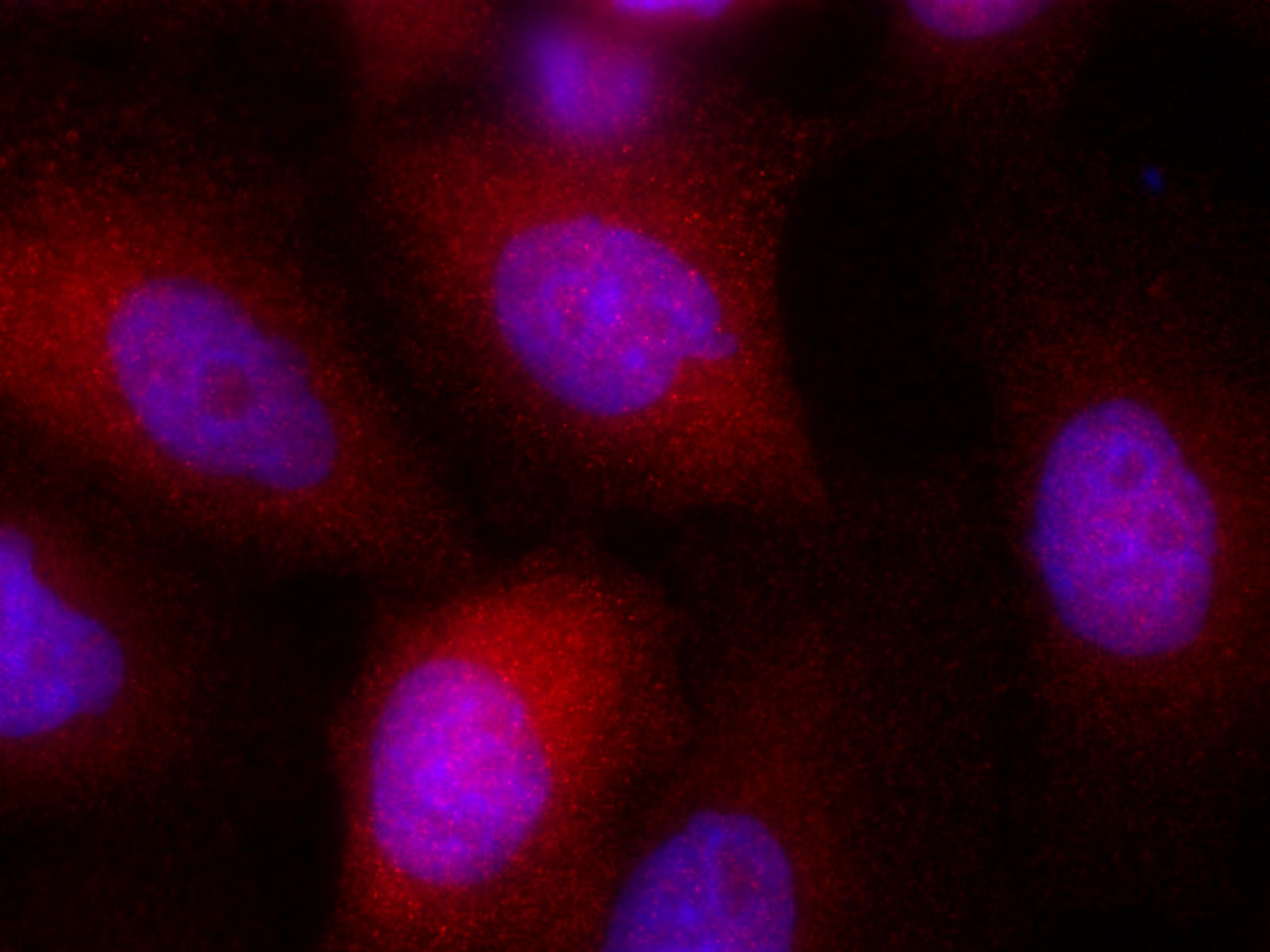

Supplement: Supplementary file 1 [file DataSheet_1.zip › Supplementary Material/IF/1.18-ERK+MITO Control 3 ^^/rb.jpg]

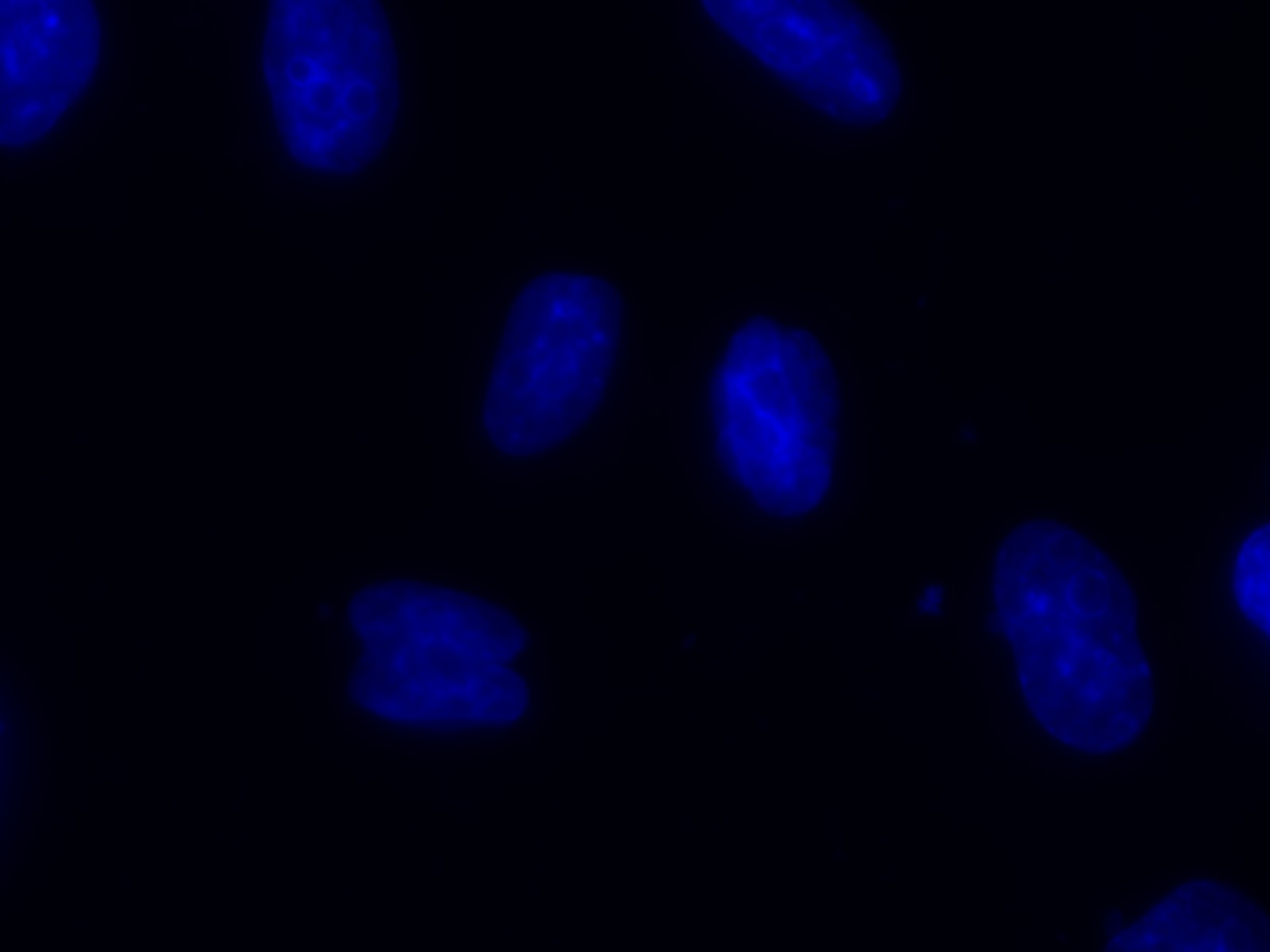

Supplement: Supplementary file 1 [file DataSheet_1.zip › Supplementary Material/IF/1.18-p-ERK+MITO GCDA+PD98059 1 ^^/b.jpg]

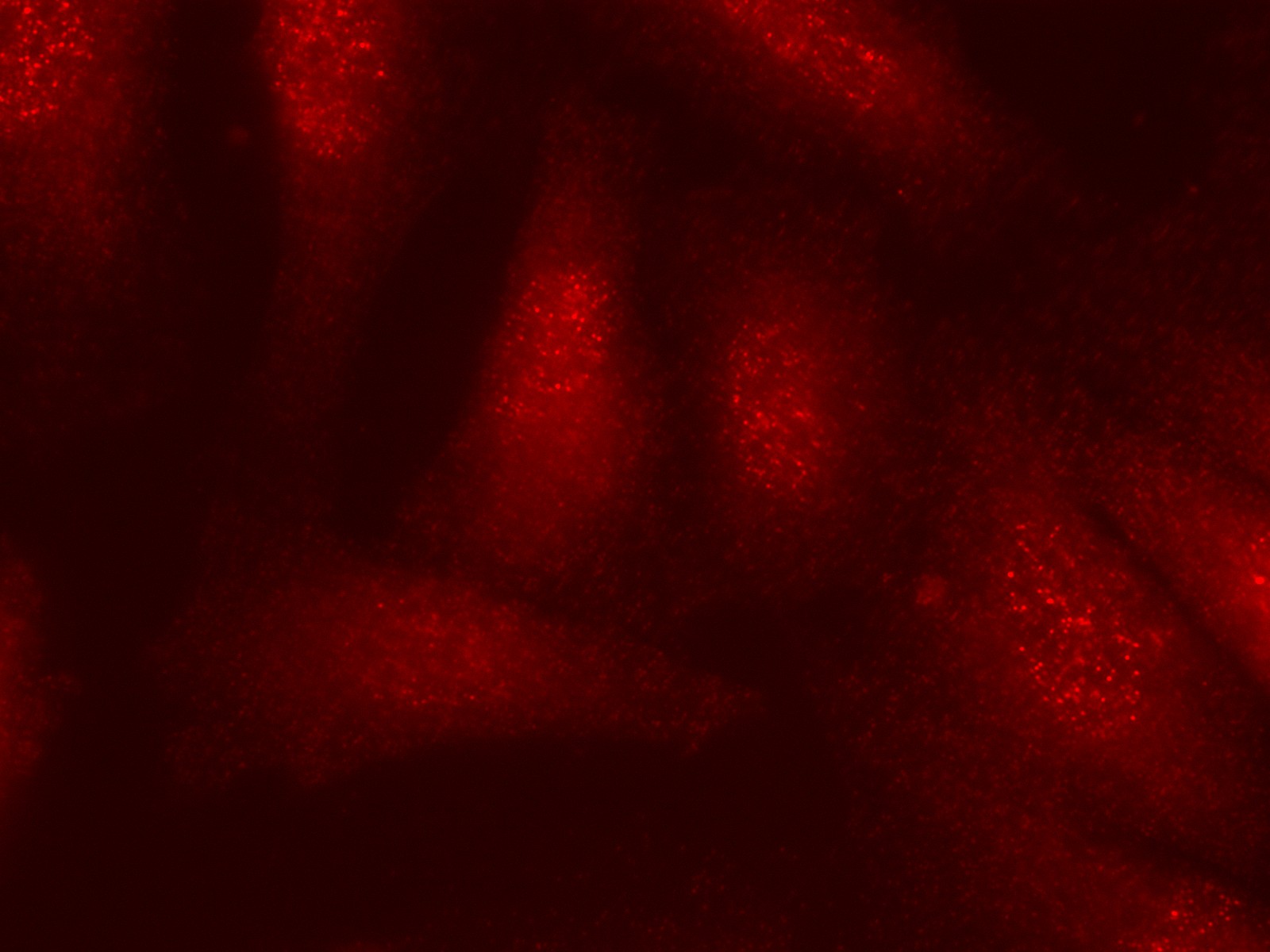

Supplement: Supplementary file 1 [file DataSheet_1.zip › Supplementary Material/IF/1.18-p-ERK+MITO GCDA+PD98059 1 ^^/r.jpg]

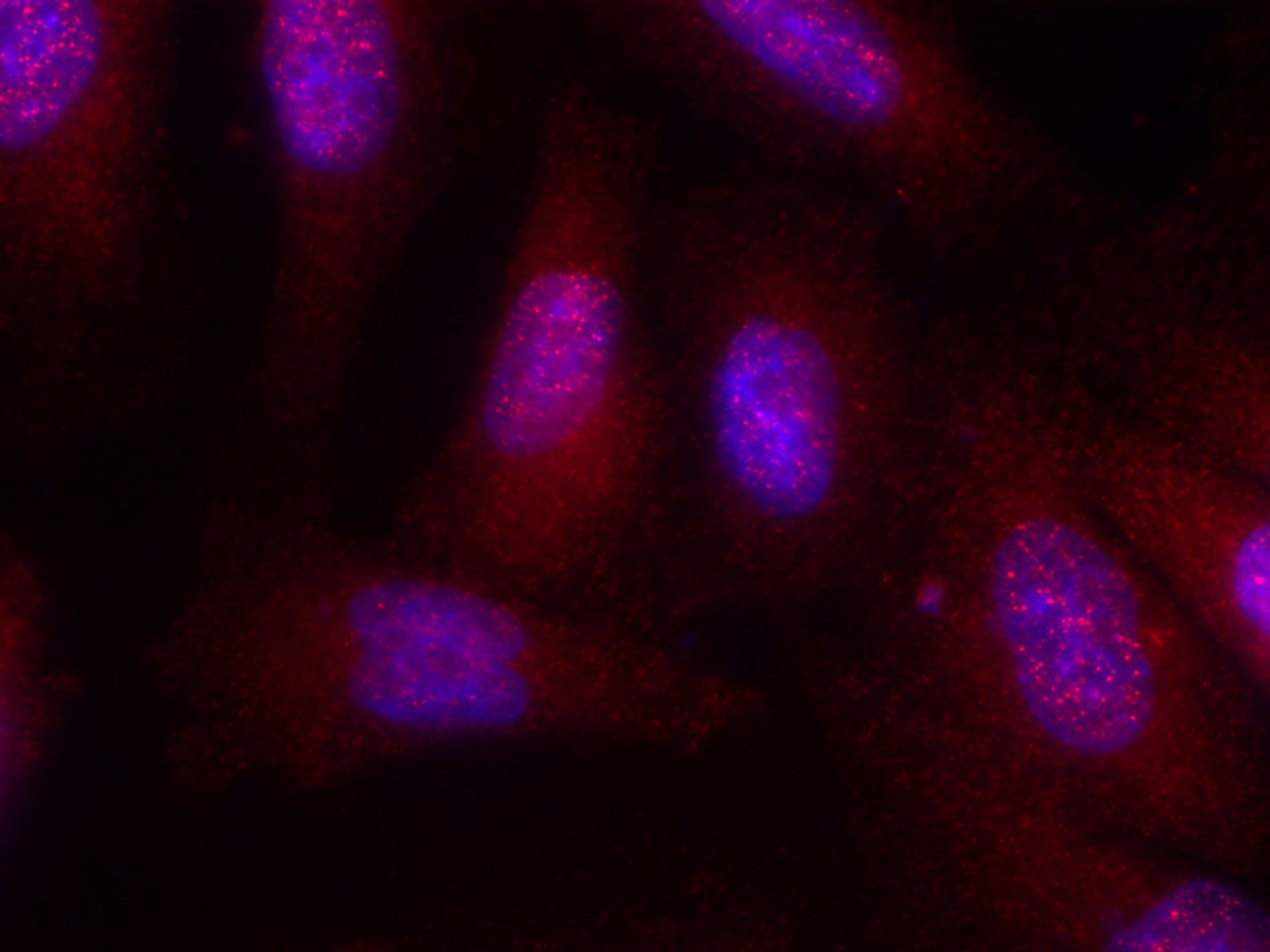

Supplement: Supplementary file 1 [file DataSheet_1.zip › Supplementary Material/IF/1.18-p-ERK+MITO GCDA+PD98059 1 ^^/rb.jpg]

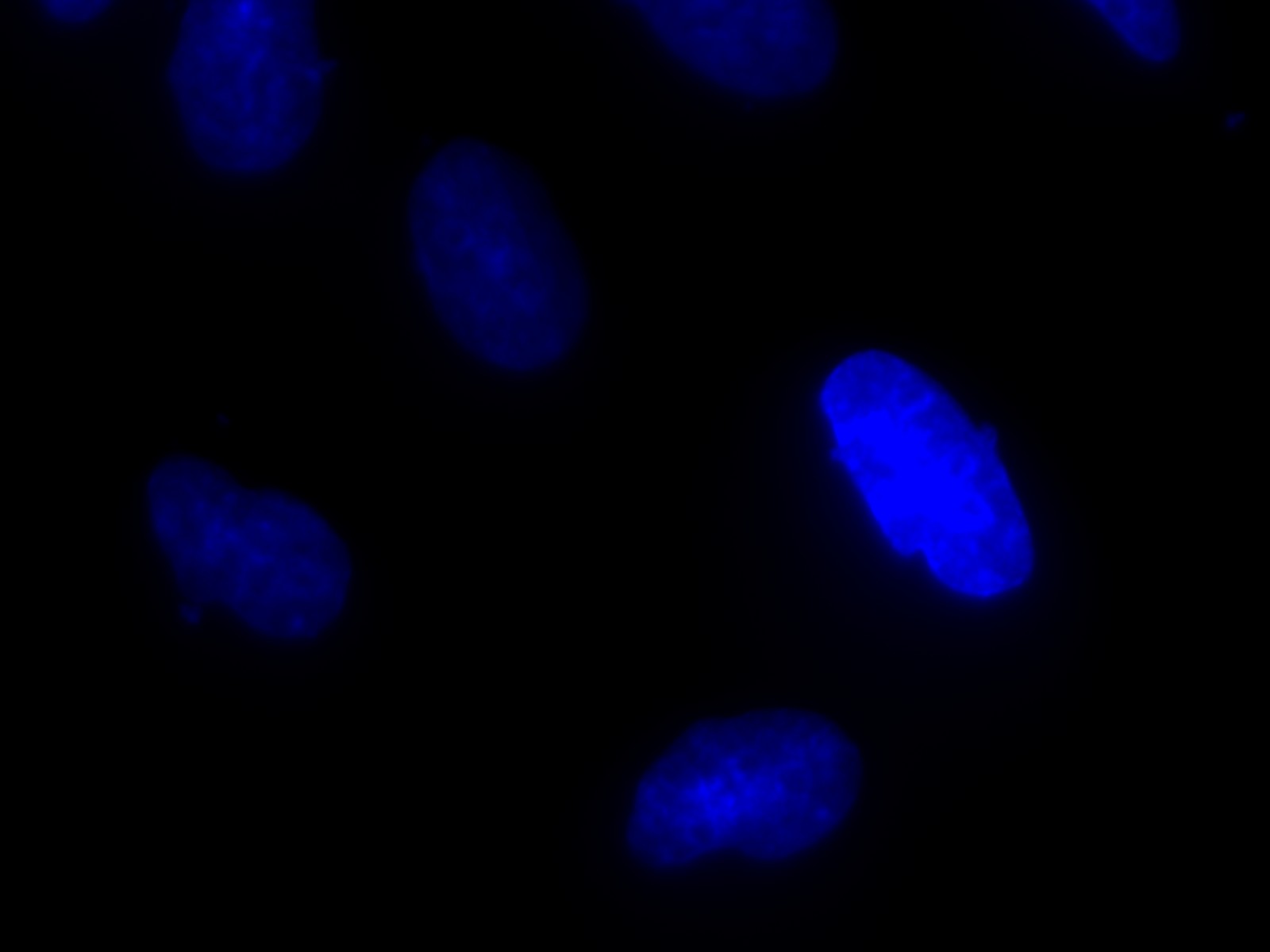

Supplement: Supplementary file 1 [file DataSheet_1.zip › Supplementary Material/IF/1.19-ERK+MITO GCDA 8h 2 ^^/b.jpg]

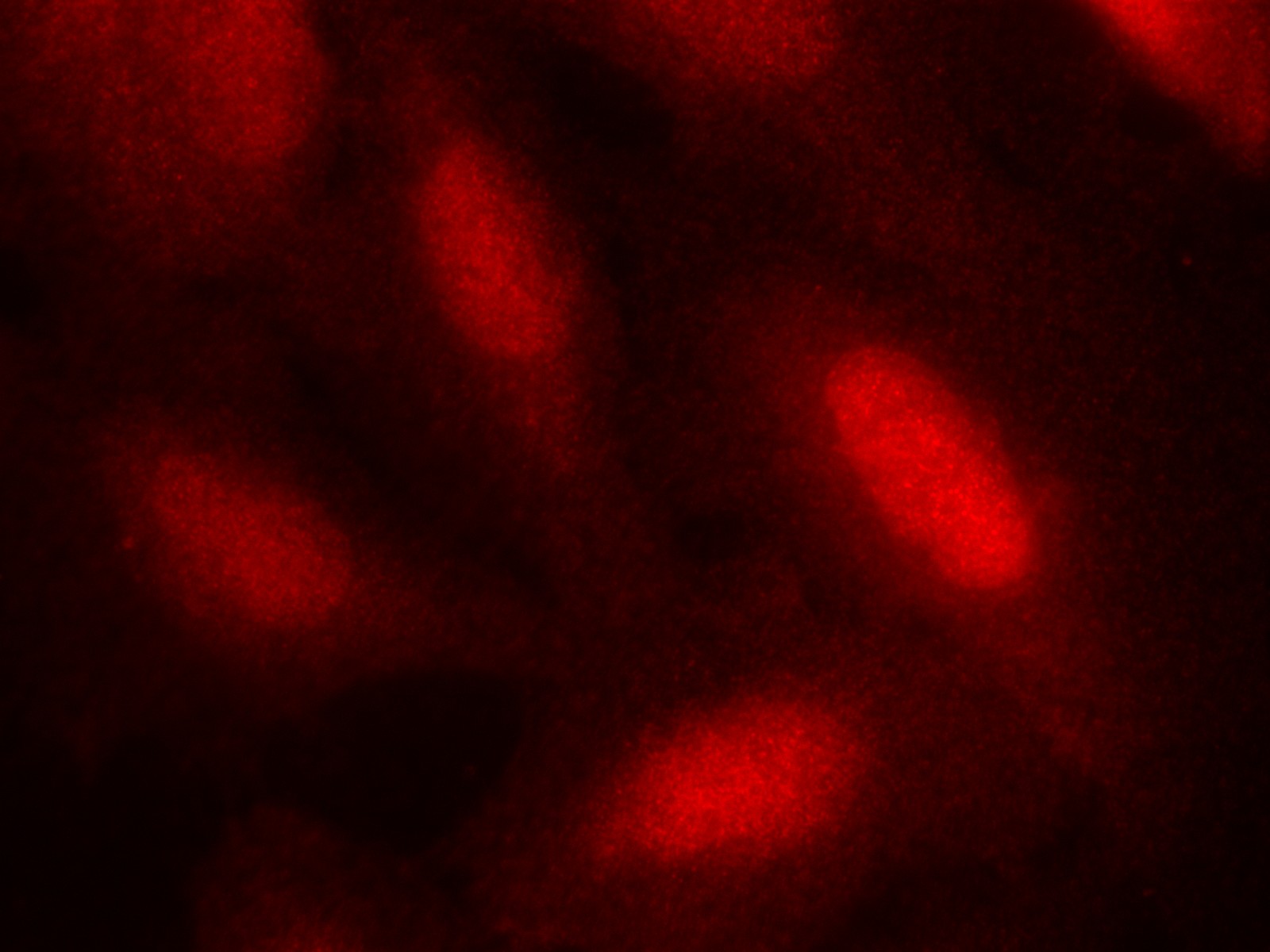

Supplement: Supplementary file 1 [file DataSheet_1.zip › Supplementary Material/IF/1.19-ERK+MITO GCDA 8h 2 ^^/r.jpg]

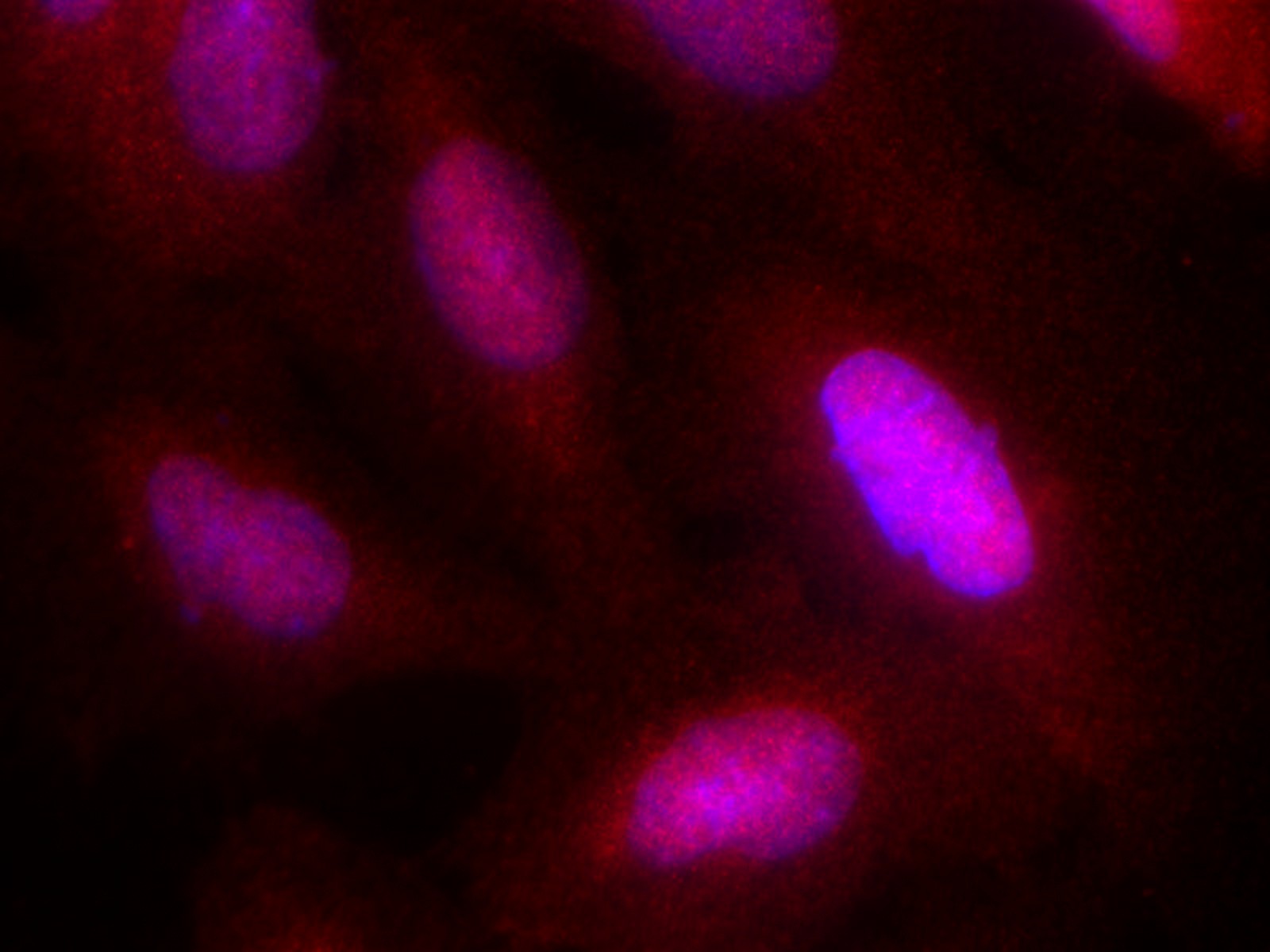

Supplement: Supplementary file 1 [file DataSheet_1.zip › Supplementary Material/IF/1.19-ERK+MITO GCDA 8h 2 ^^/rb.jpg]

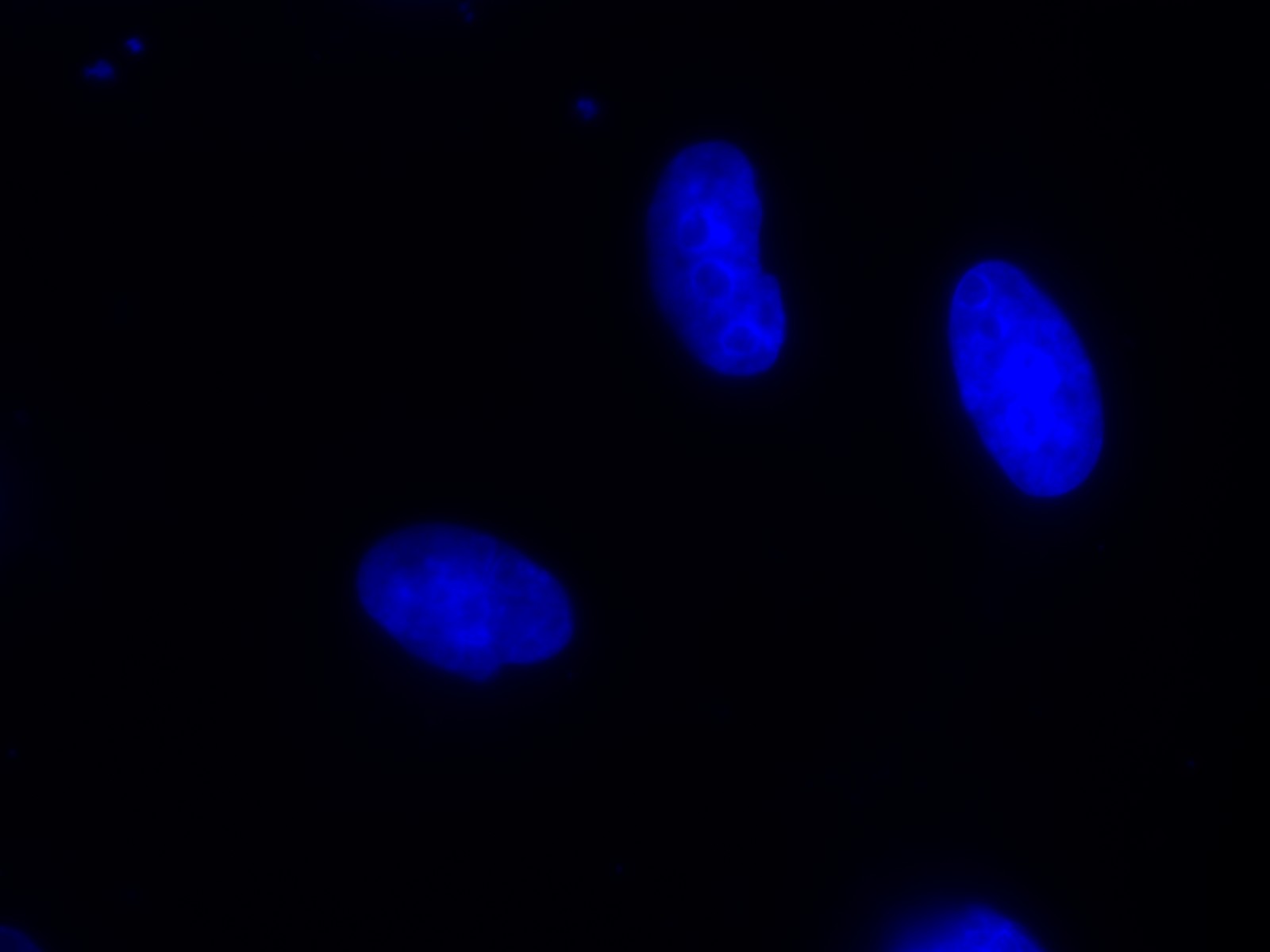

Supplement: Supplementary file 1 [file DataSheet_1.zip › Supplementary Material/IF/1.19-ERK+MITO GCDA+PD98059 1 ^^/b.jpg]

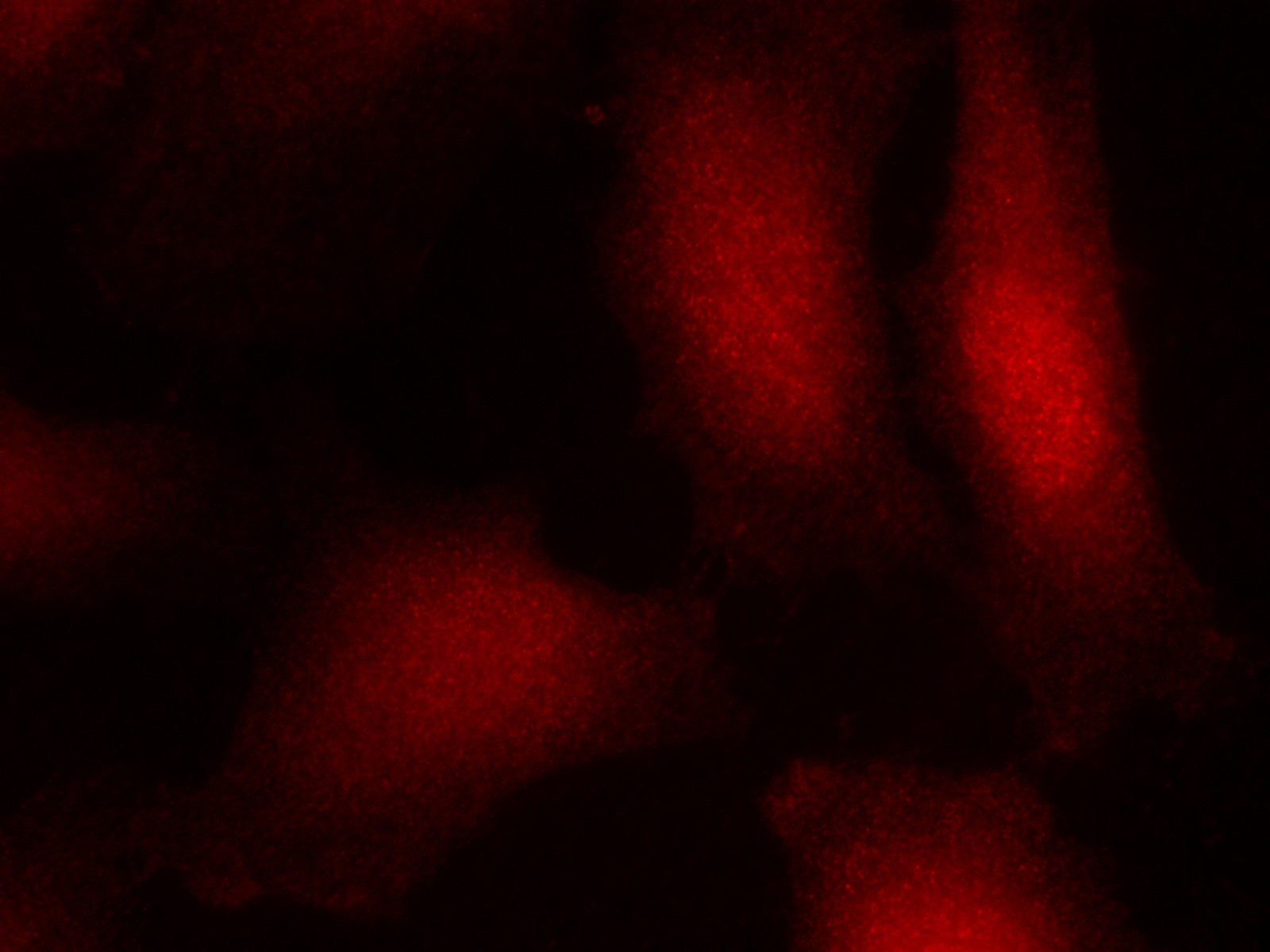

Supplement: Supplementary file 1 [file DataSheet_1.zip › Supplementary Material/IF/1.19-ERK+MITO GCDA+PD98059 1 ^^/r.jpg]

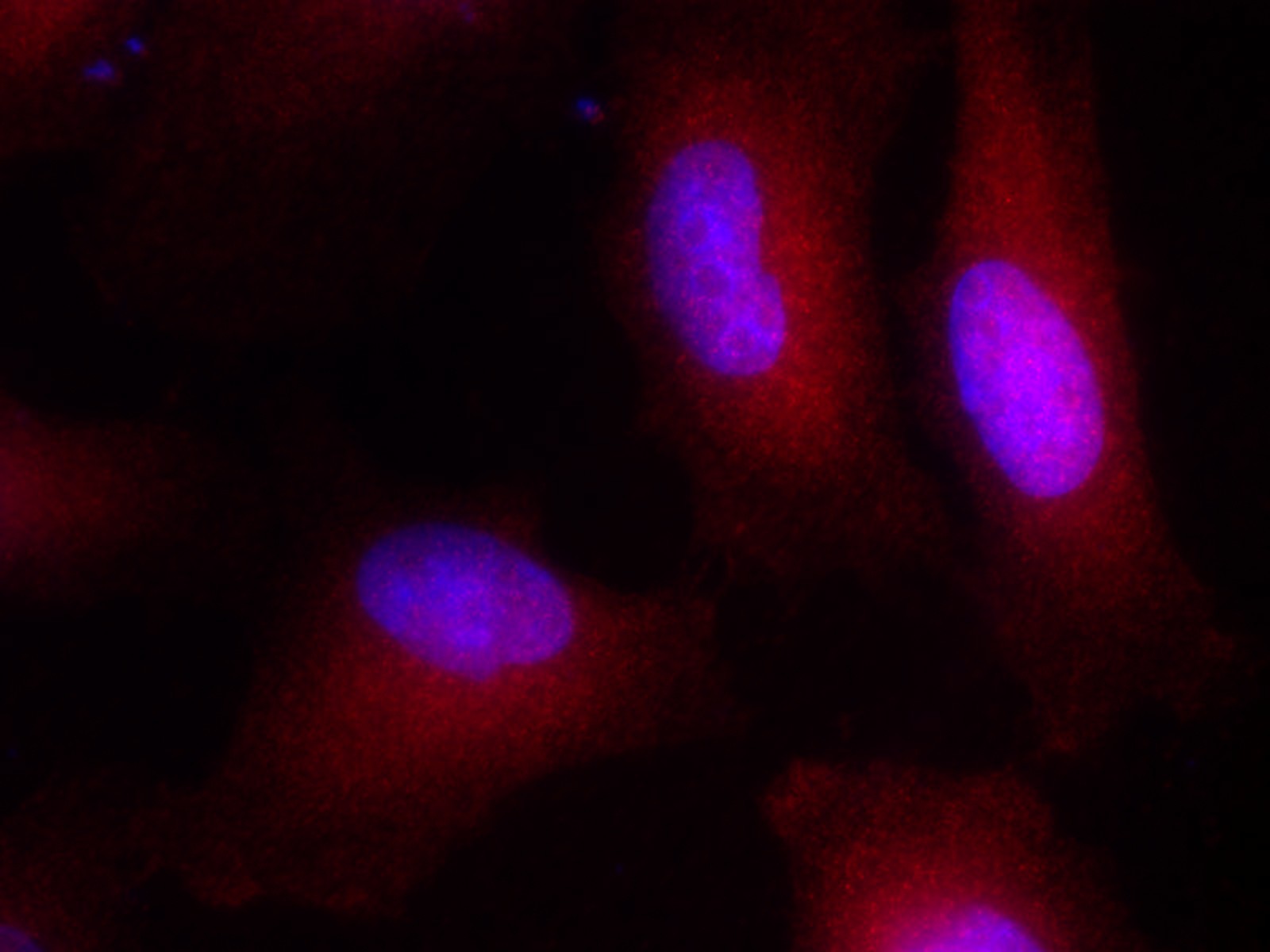

Supplement: Supplementary file 1 [file DataSheet_1.zip › Supplementary Material/IF/1.19-ERK+MITO GCDA+PD98059 1 ^^/rb.jpg]

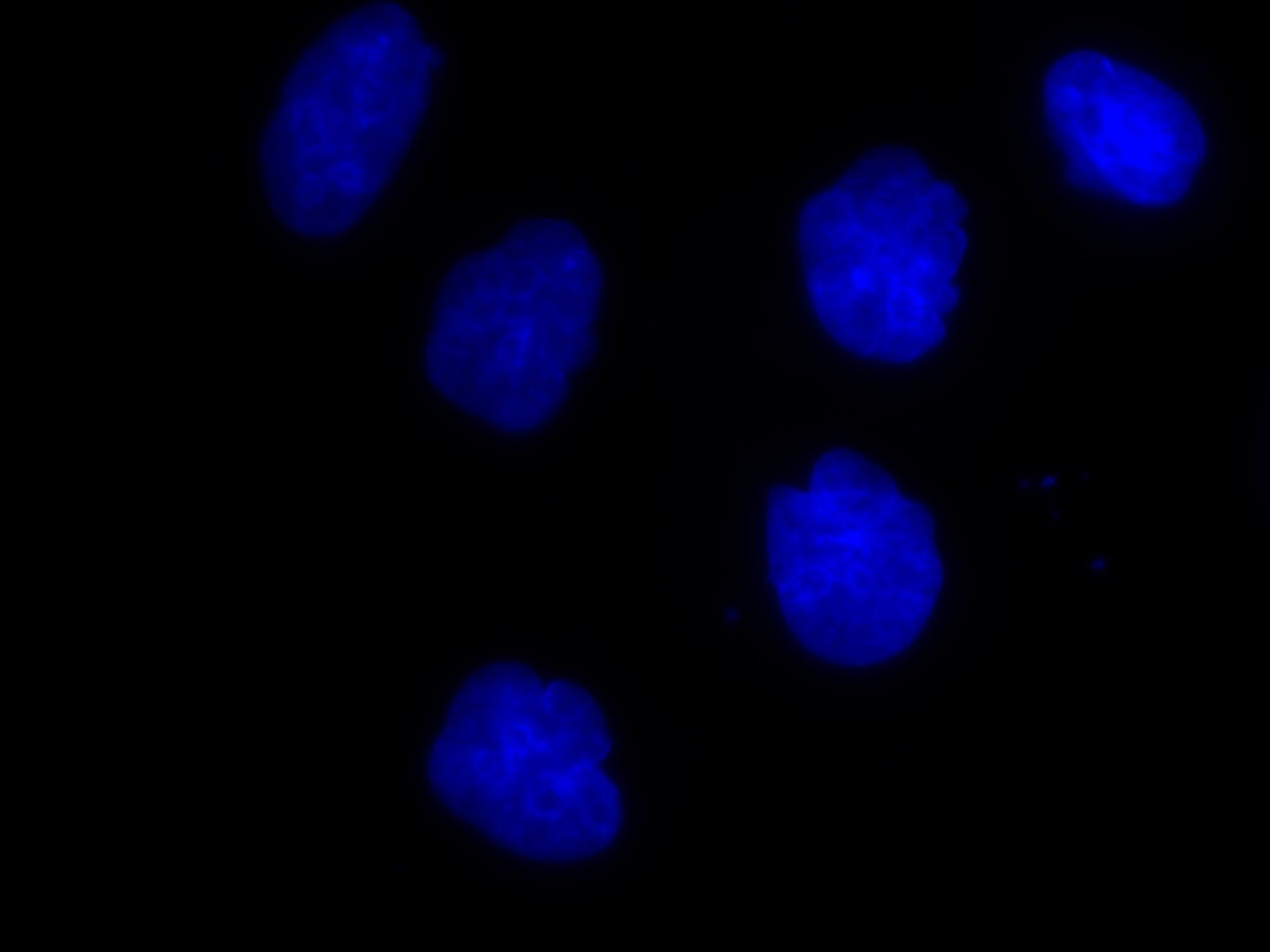

Supplement: Supplementary file 1 [file DataSheet_1.zip › Supplementary Material/IF/1.19-p-ERK+MITO Control 2 ^^/b.jpg]

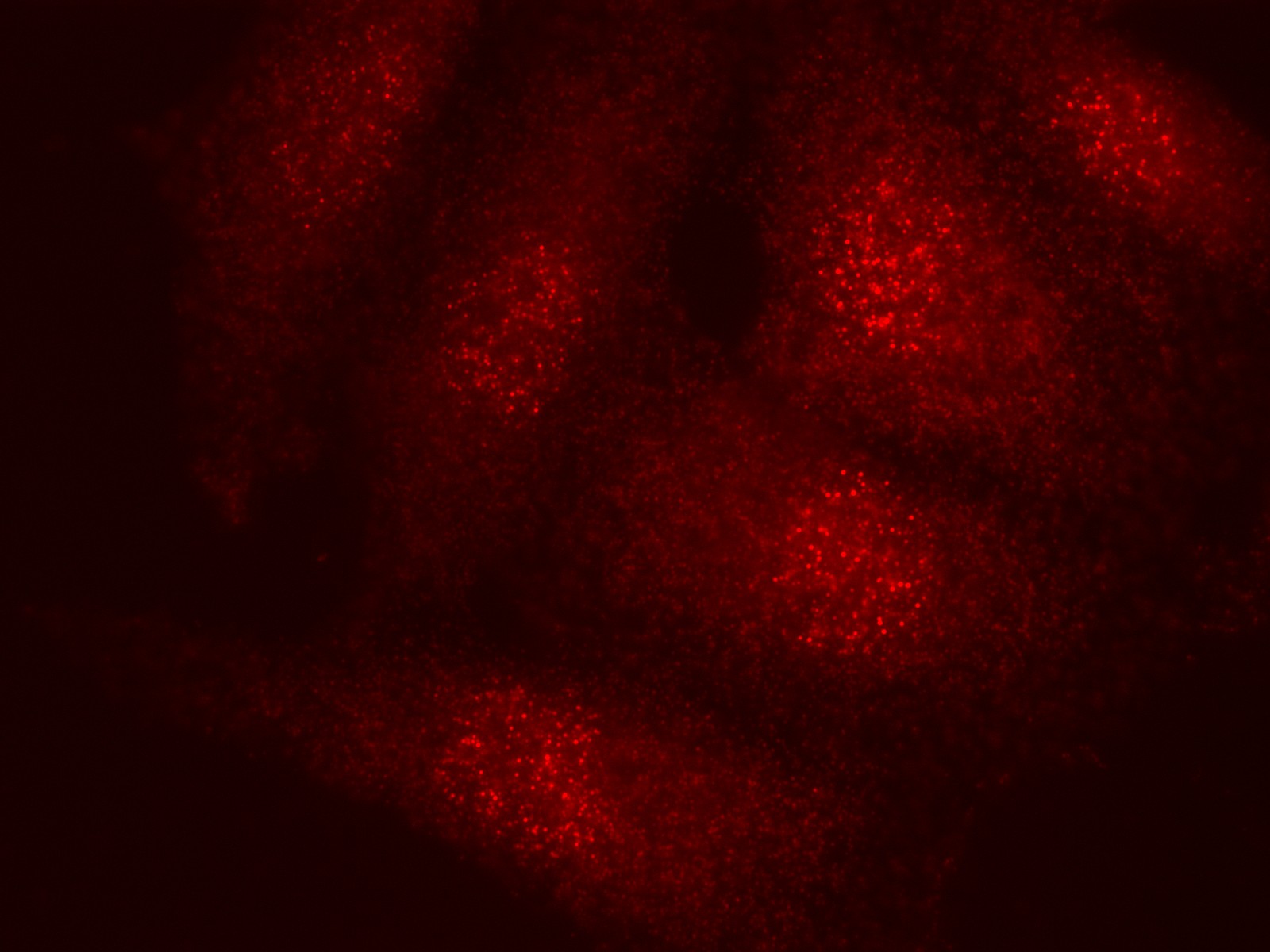

Supplement: Supplementary file 1 [file DataSheet_1.zip › Supplementary Material/IF/1.19-p-ERK+MITO Control 2 ^^/r.jpg]

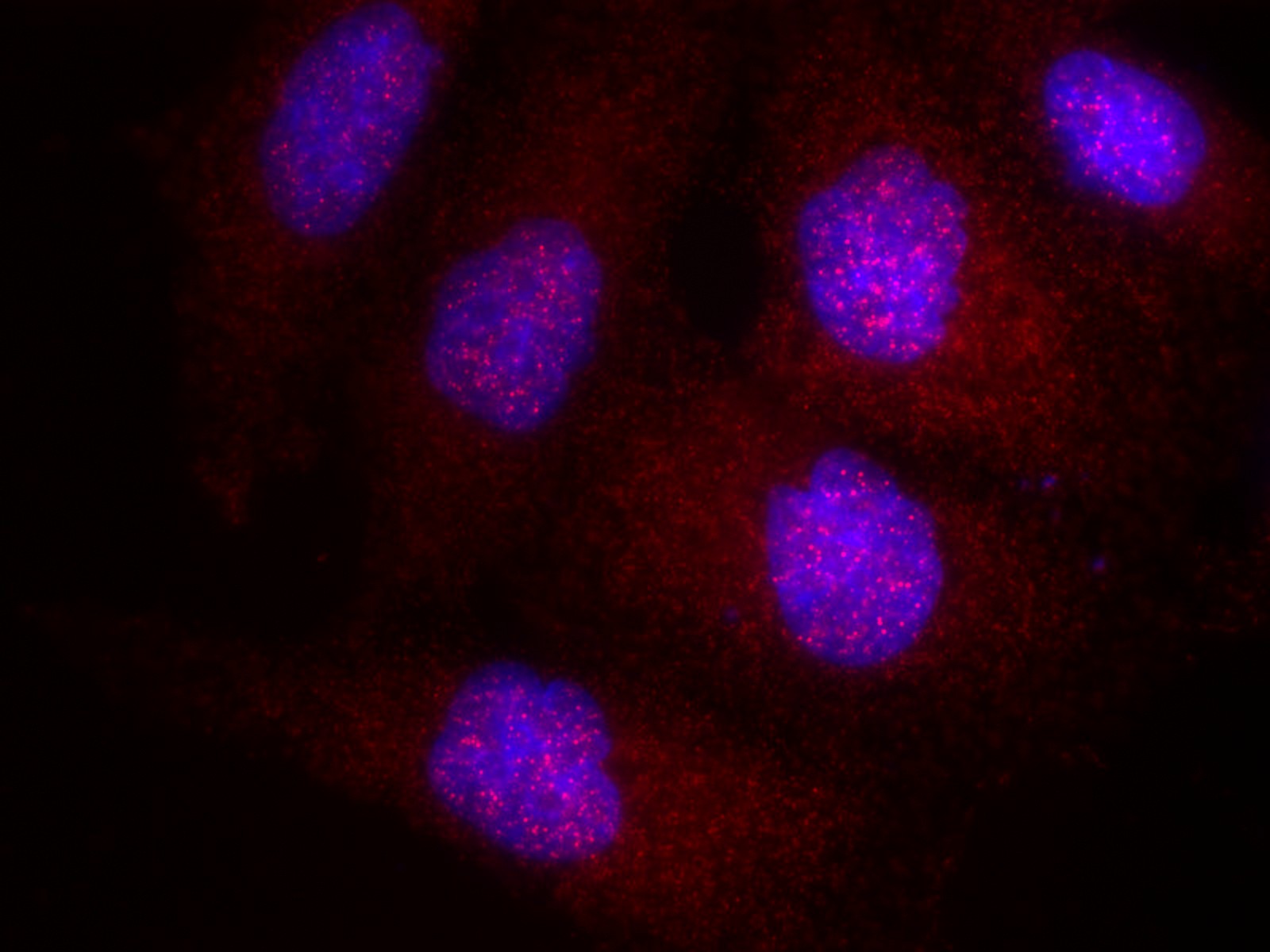

Supplement: Supplementary file 1 [file DataSheet_1.zip › Supplementary Material/IF/1.19-p-ERK+MITO Control 2 ^^/rb.jpg]

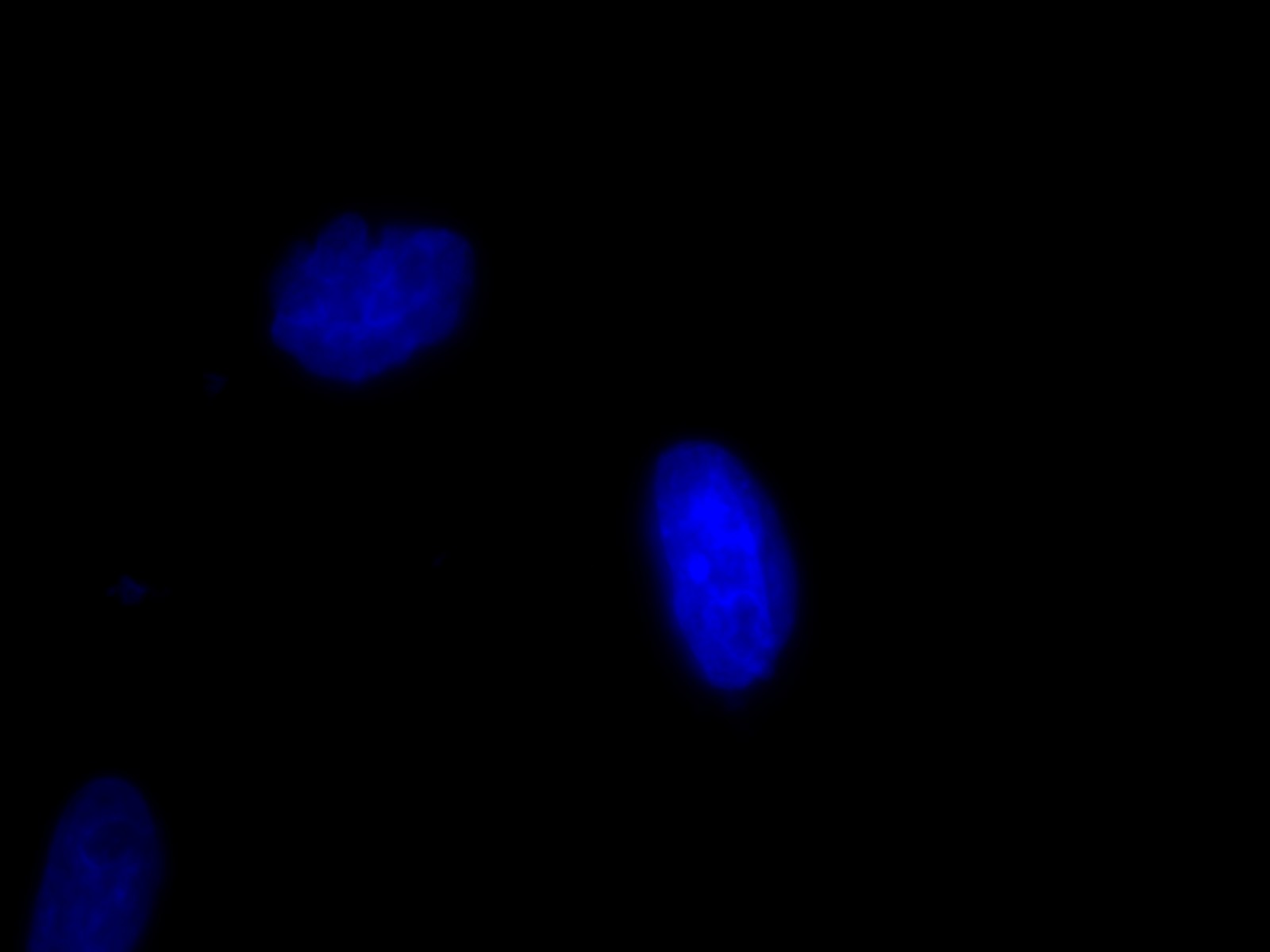

Supplement: Supplementary file 1 [file DataSheet_1.zip › Supplementary Material/IF/1.19-p-ERK+MITO GCDA 8h 2 ^^/b.jpg]

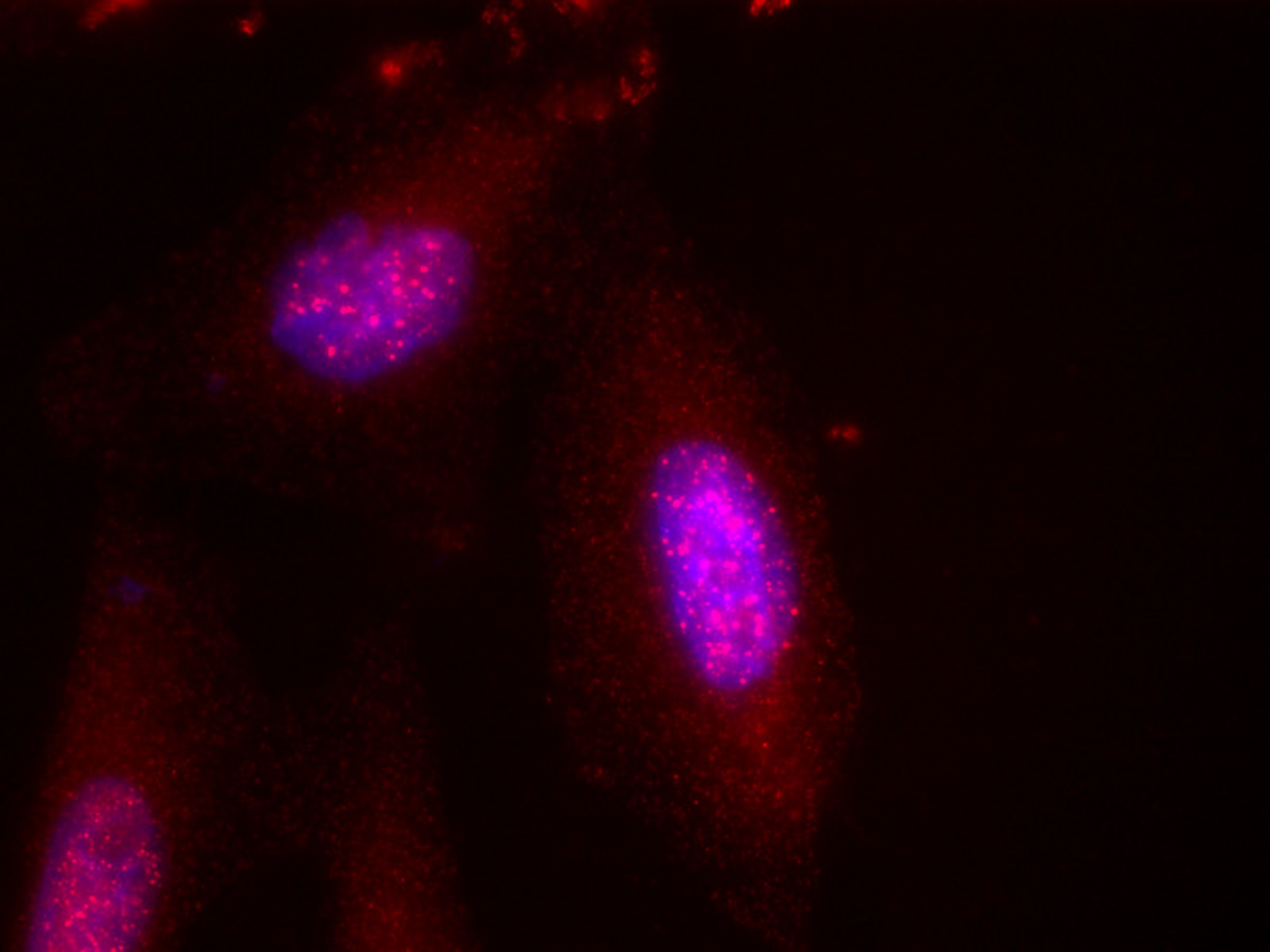

Supplement: Supplementary file 1 [file DataSheet_1.zip › Supplementary Material/IF/1.19-p-ERK+MITO GCDA 8h 2 ^^/r b.jpg]

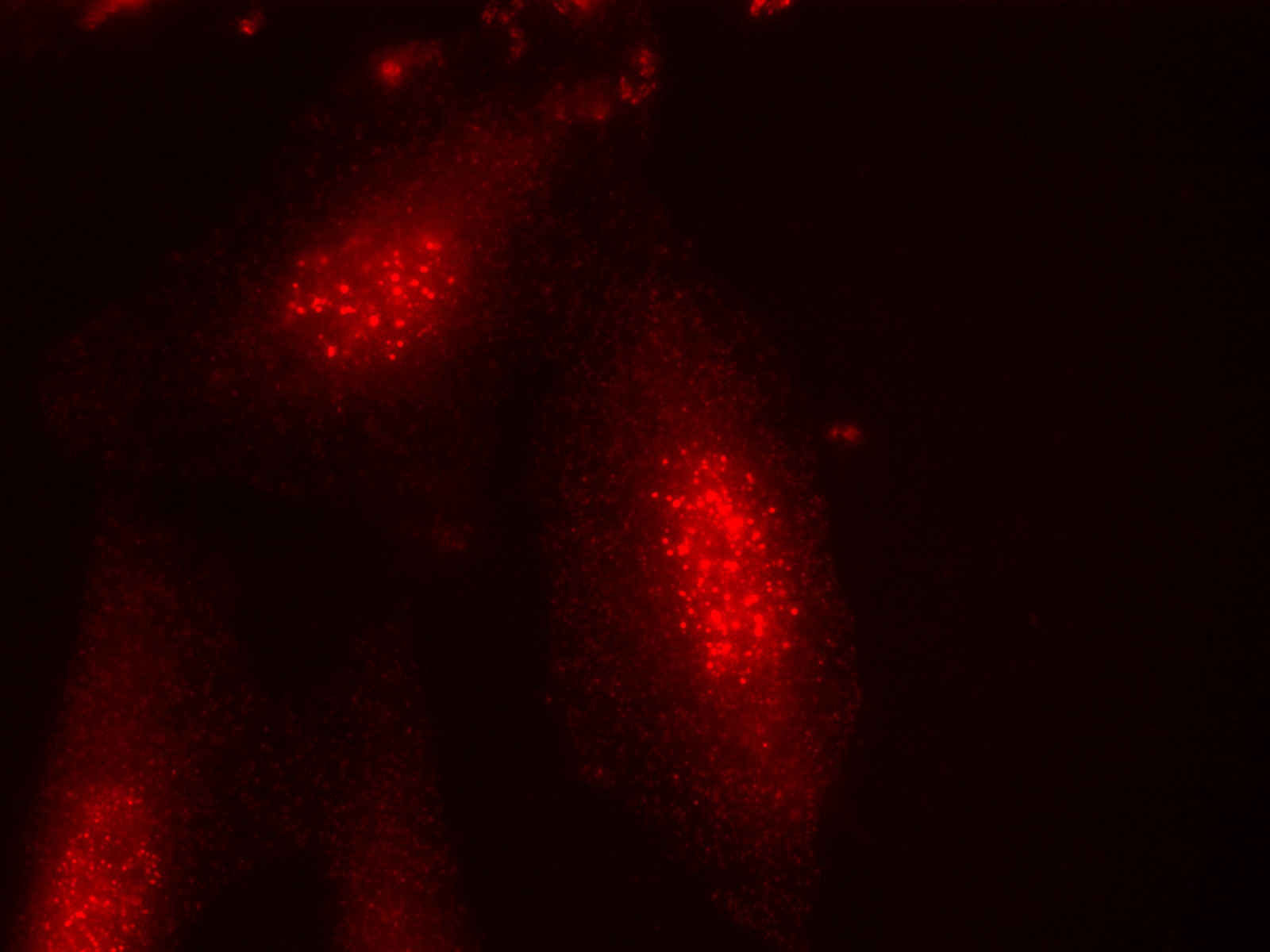

Supplement: Supplementary file 1 [file DataSheet_1.zip › Supplementary Material/IF/1.19-p-ERK+MITO GCDA 8h 2 ^^/r.jpg]

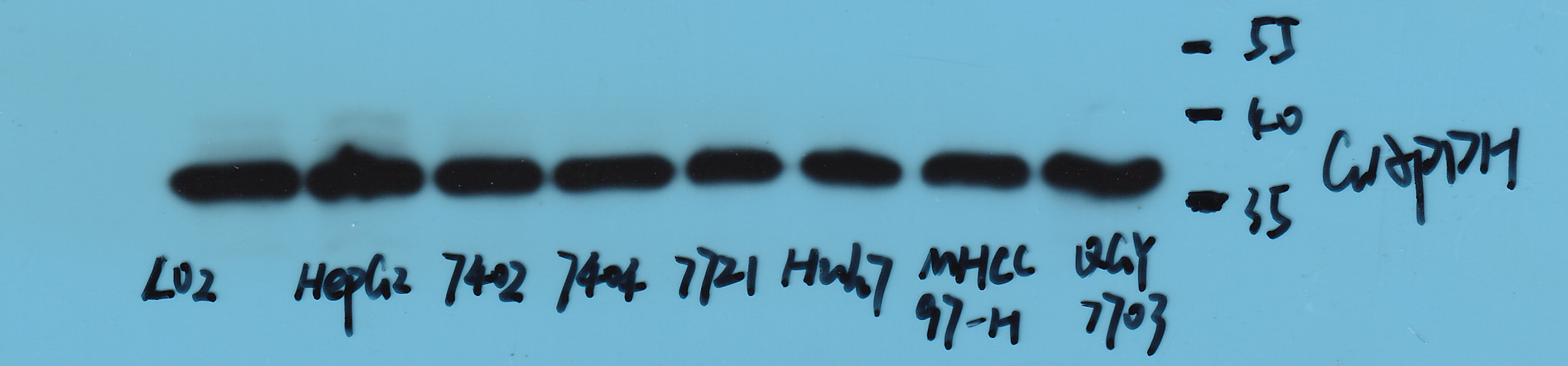

Supplement: Supplementary file 1 [file DataSheet_1.zip › Supplementary Material/western blot/Figure 1/Figure 1A-GAPDH.tif]

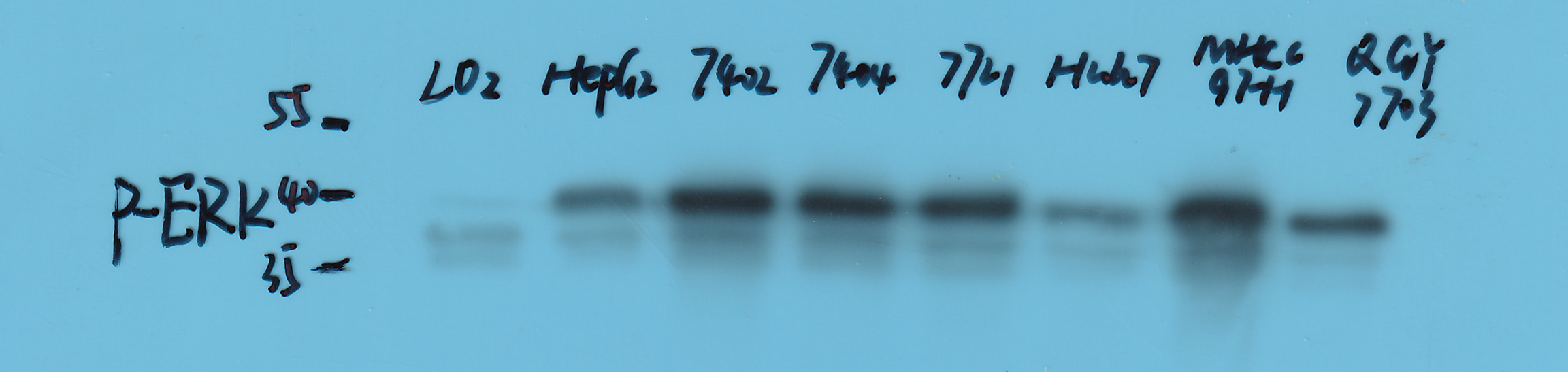

Supplement: Supplementary file 1 [file DataSheet_1.zip › Supplementary Material/western blot/Figure 1/Figure 1A-P-ERK.tif]

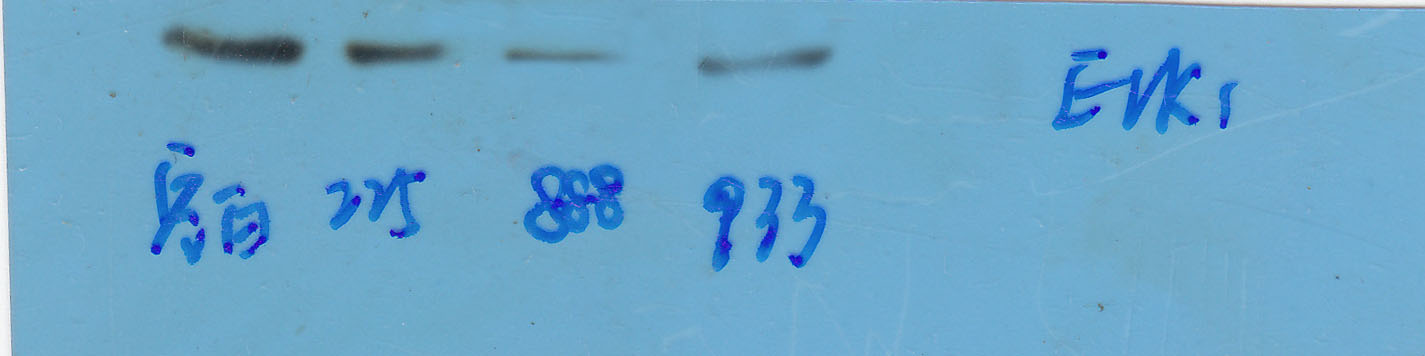

Supplement: Supplementary file 1 [file DataSheet_1.zip › Supplementary Material/western blot/Figure 1/Figure 1C-ERK1.jpg]

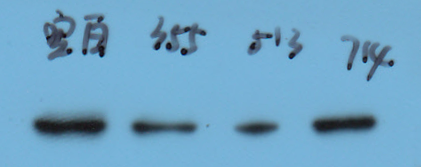

Supplement: Supplementary file 1 [file DataSheet_1.zip › Supplementary Material/western blot/Figure 1/Figure 1C-ERK2.tif]

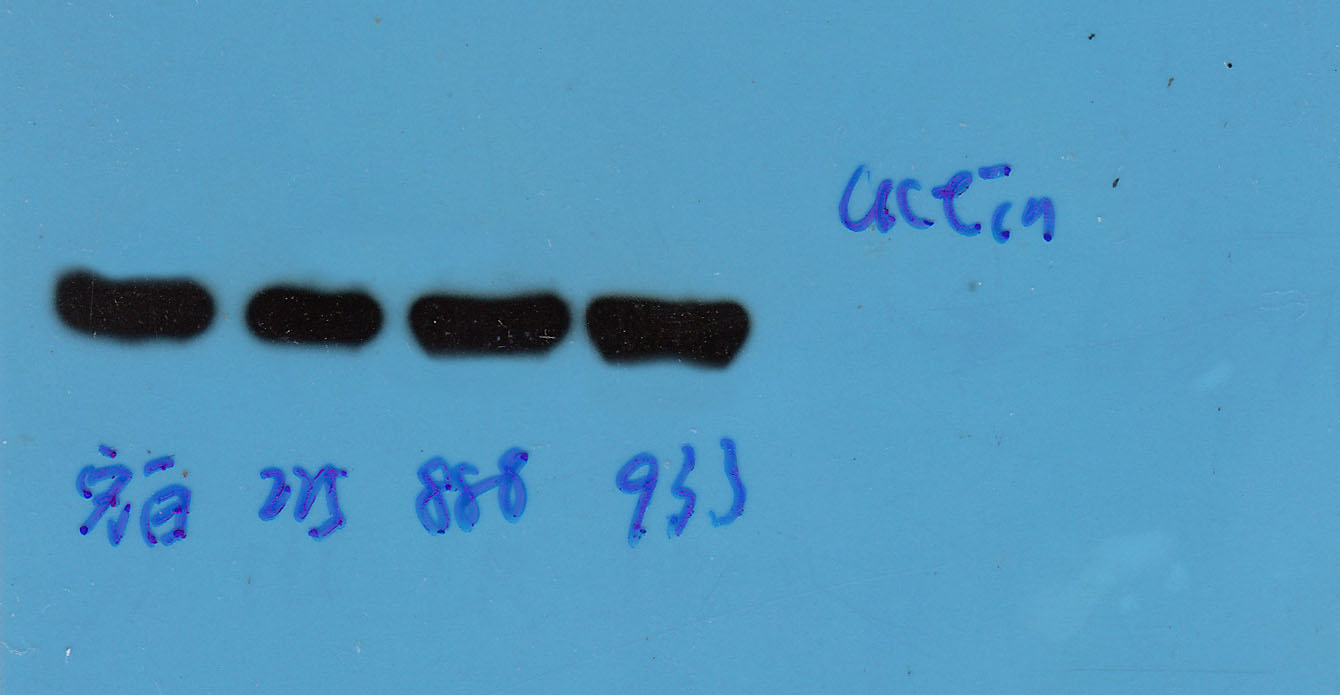

Supplement: Supplementary file 1 [file DataSheet_1.zip › Supplementary Material/western blot/Figure 1/Figure 1C-a┬-Actin(ERK1).jpg]

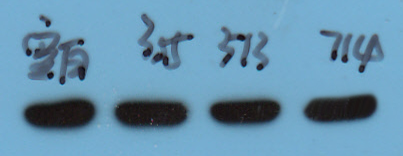

Supplement: Supplementary file 1 [file DataSheet_1.zip › Supplementary Material/western blot/Figure 1/Figure 1C-a┬-Actin(ERK2).tif]

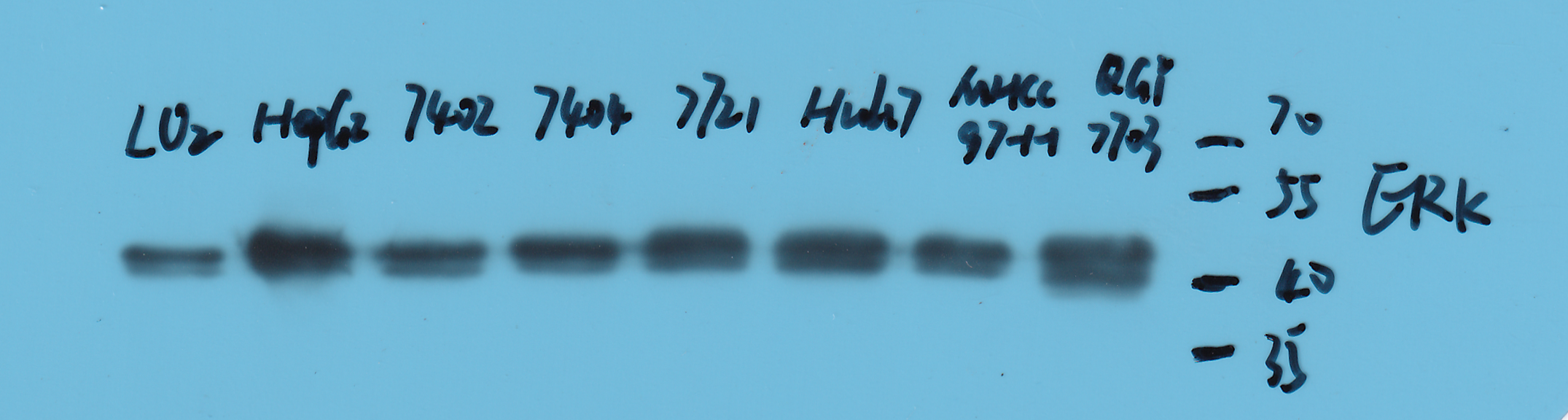

Supplement: Supplementary file 1 [file DataSheet_1.zip › Supplementary Material/western blot/Figure 1/Figure1A-ERK.tif]

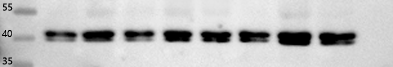

Supplement: Supplementary file 1 [file DataSheet_1.zip › Supplementary Material/western blot/Figure 2/Figure 2A-WB/ERK+marker.tif]

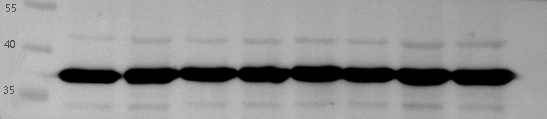

Supplement: Supplementary file 1 [file DataSheet_1.zip › Supplementary Material/western blot/Figure 2/Figure 2A-WB/GAPDH+marker.tif]

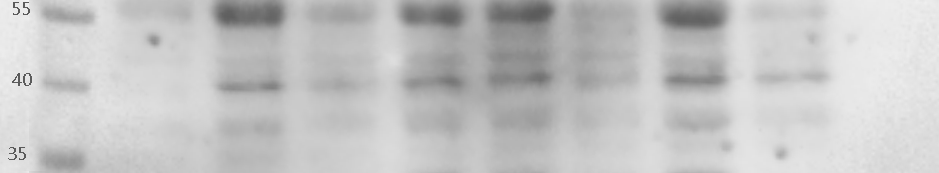

Supplement: Supplementary file 1 [file DataSheet_1.zip › Supplementary Material/western blot/Figure 2/Figure 2A-WB/P-ERK+marker.tif]

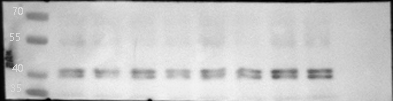

Supplement: Supplementary file 1 [file DataSheet_1.zip › Supplementary Material/western blot/Figure 2/Figure 2B-WB/ERK.tif]

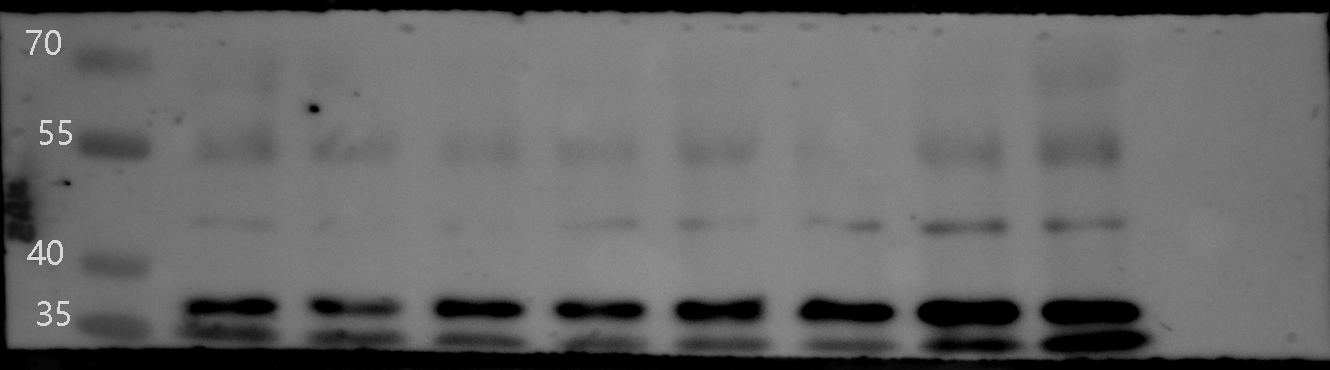

Supplement: Supplementary file 1 [file DataSheet_1.zip › Supplementary Material/western blot/Figure 2/Figure 2B-WB/GAPDH-ERK.tif]

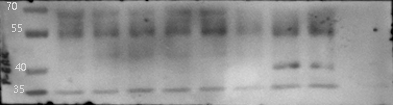

Supplement: Supplementary file 1 [file DataSheet_1.zip › Supplementary Material/western blot/Figure 2/Figure 2B-WB/P-ERK.tif]

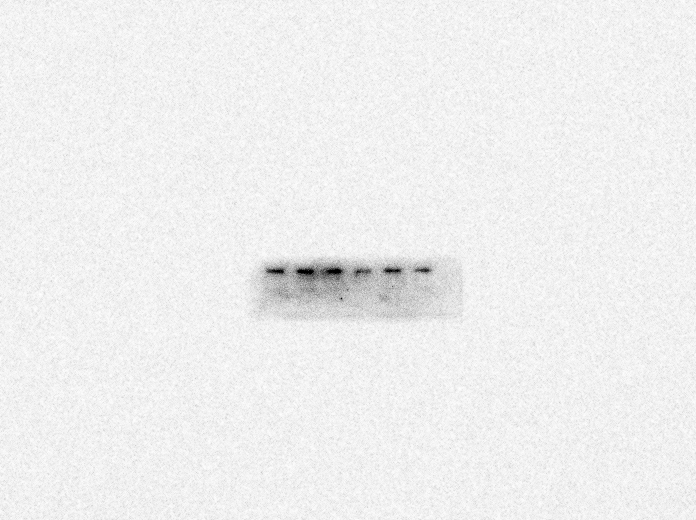

Supplement: Supplementary file 1 [file DataSheet_1.zip › Supplementary Material/western blot/Figure 5/Figure 5A-BAK.tif]

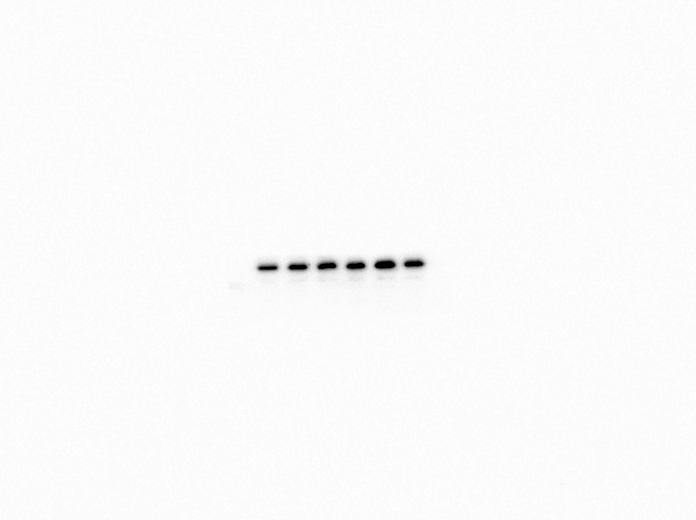

Supplement: Supplementary file 1 [file DataSheet_1.zip › Supplementary Material/western blot/Figure 5/Figure 5A-Bcl2.tif]

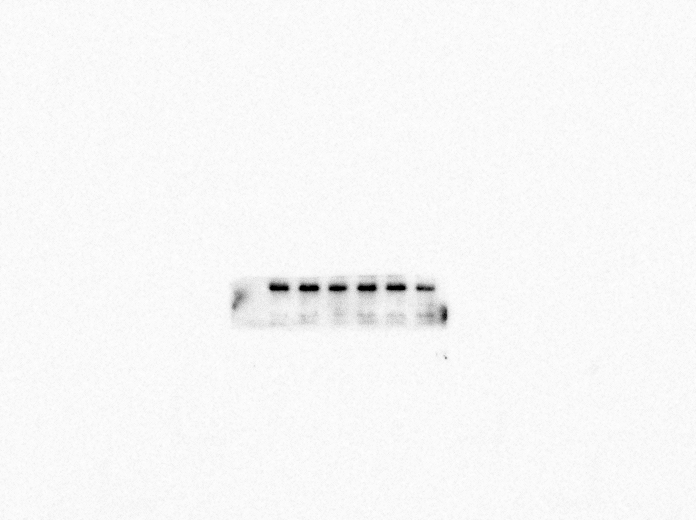

Supplement: Supplementary file 1 [file DataSheet_1.zip › Supplementary Material/western blot/Figure 5/Figure 5A-Bim.tif]

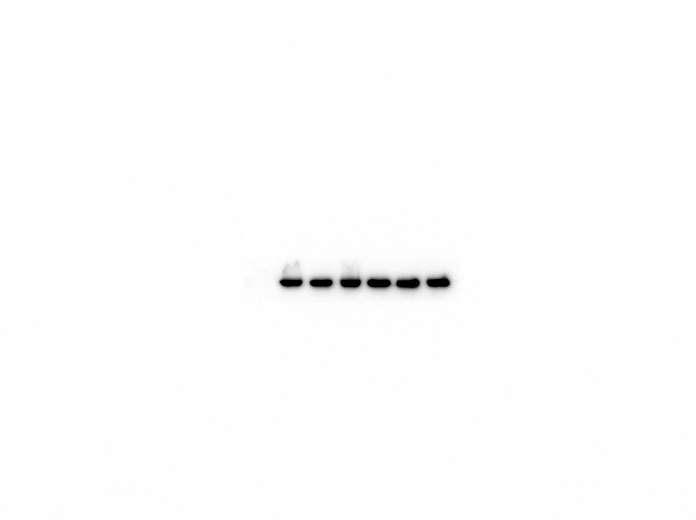

Supplement: Supplementary file 1 [file DataSheet_1.zip › Supplementary Material/western blot/Figure 5/Figure 5A-GAPDH.tif]

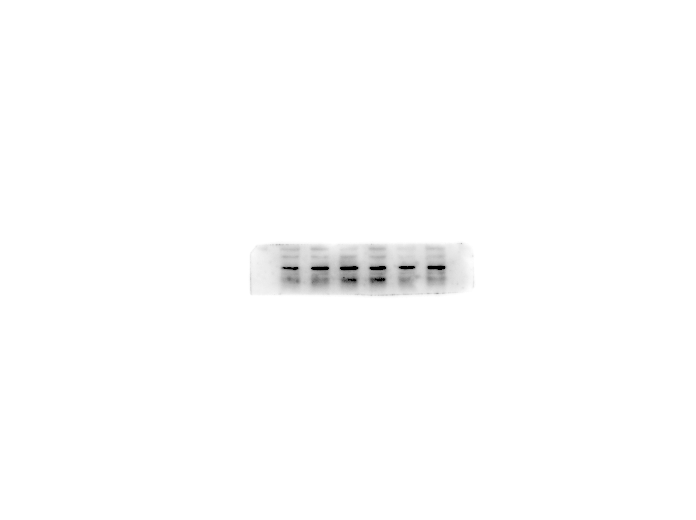

Supplement: Supplementary file 1 [file DataSheet_1.zip › Supplementary Material/western blot/Figure 5/Figure 5A-Mcl1.tif]

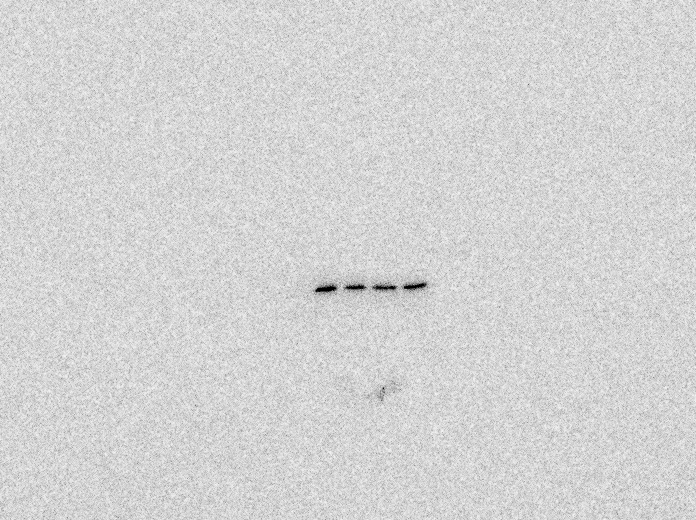

Supplement: Supplementary file 1 [file DataSheet_1.zip › Supplementary Material/western blot/Figure 5/Figure 5B-Bak.tif]

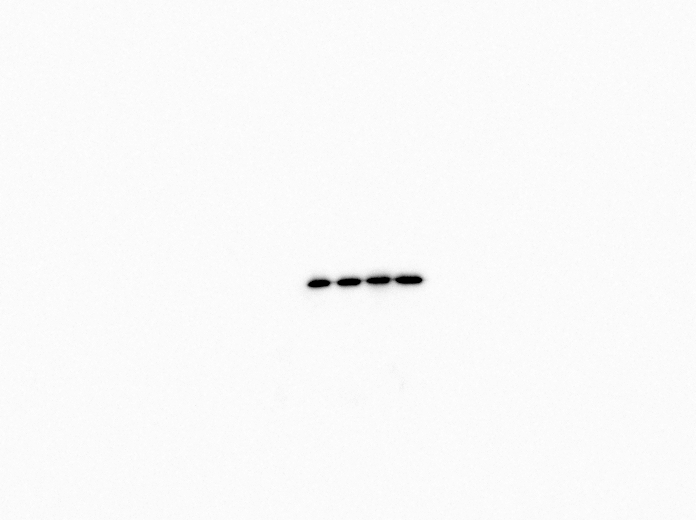

Supplement: Supplementary file 1 [file DataSheet_1.zip › Supplementary Material/western blot/Figure 5/Figure 5B-Bcl2.tif]

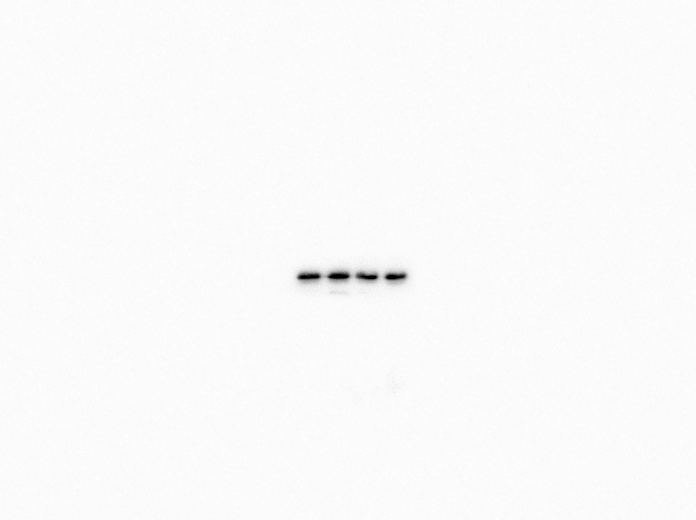

Supplement: Supplementary file 1 [file DataSheet_1.zip › Supplementary Material/western blot/Figure 5/Figure 5B-Bim.tif]

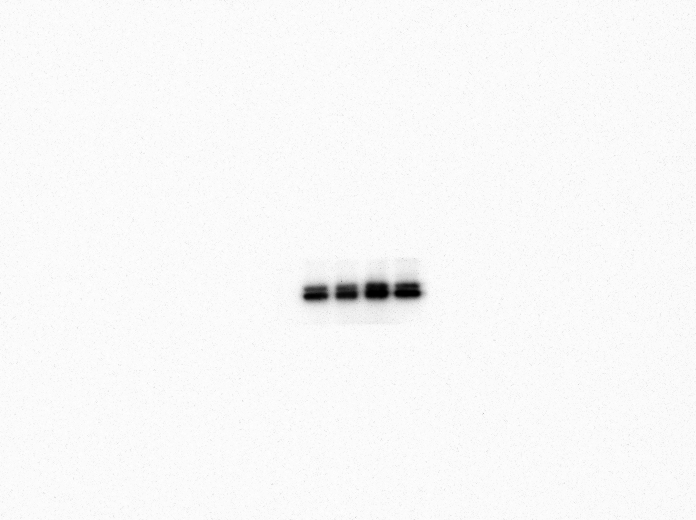

Supplement: Supplementary file 1 [file DataSheet_1.zip › Supplementary Material/western blot/Figure 5/Figure 5B-ERK.tif]

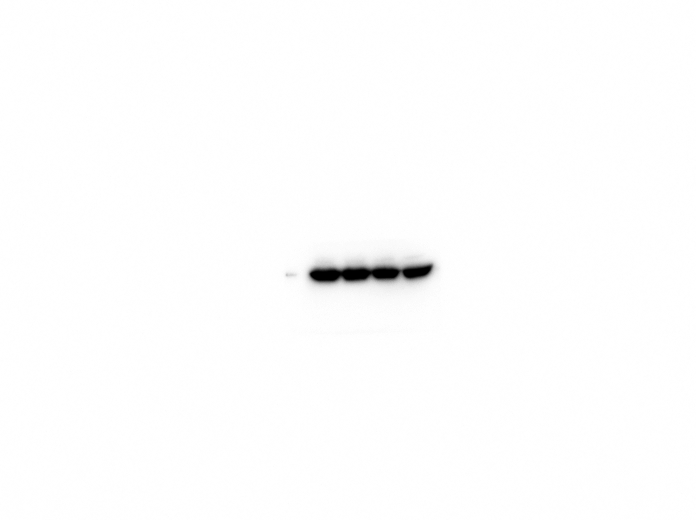

Supplement: Supplementary file 1 [file DataSheet_1.zip › Supplementary Material/western blot/Figure 5/Figure 5B-GAPDH.tif]

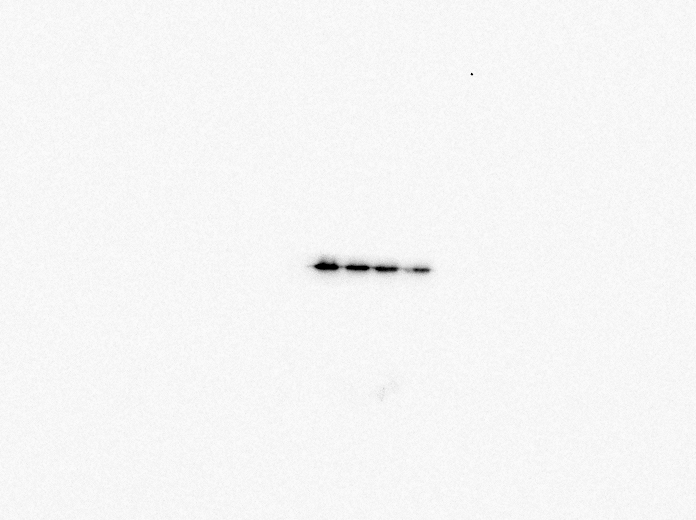

Supplement: Supplementary file 1 [file DataSheet_1.zip › Supplementary Material/western blot/Figure 5/Figure 5B-Mcl1.tif]

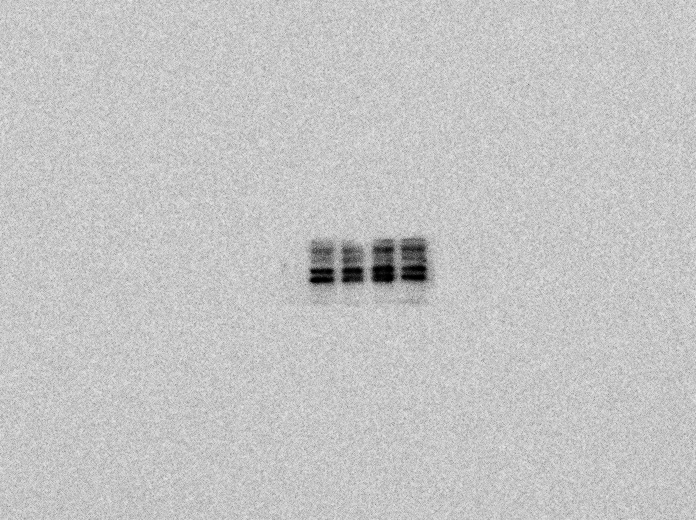

Supplement: Supplementary file 1 [file DataSheet_1.zip › Supplementary Material/western blot/Figure 5/Figure 5B-p-ERK.tif]
